# Reassessment of French breeding bird population sizes using citizen science and accounting for species detectability

**DOI:** 10.7717/peerj.17889

**Published:** 2024-08-27

**Authors:** Jean Nabias, Luc Barbaro, Benoît Fontaine, Jérémy Dupuy, Laurent Couzi, Clément Vallé, Romain Lorrilliere

**Affiliations:** 1 Muséum National d’Histoire Naturelle, Centre d’Ecologie et des Sciences de la Conservation, Paris, France; 2 Ligue Pour la Protection des Oiseaux, Rochefort, France; 3 Institut National de la Recherche pour l’Agriculture, l’Alimentation et l’Environnement (INRAE), Auzeville-Tolosane, France; 4 Patrimoine Naturel, Office Français de la Biodiversité, Paris, France; 5 Centre de Recherches sur la Biologie des Populations d’Oiseaux, Paris, France

**Keywords:** Bird atlases, Biogeography, Breeding bird surveys, Citizen science, Detectability, Hierarchical distance sampling

## Abstract

Higher efficiency in large-scale and long-term biodiversity monitoring can be obtained through the use of Essential Biodiversity Variables, among which species population sizes provide key data for conservation programs. Relevant estimations and assessment of actual population sizes are critical for species conservation, especially in the current context of global biodiversity erosion. However, knowledge on population size varies greatly, depending on species conservation status and ranges. While the most threatened or restricted-range species generally benefit from exhaustive counts and surveys, monitoring common and widespread species population size tends to be neglected or is simply more challenging to achieve. In such a context, citizen science (CS) is a powerful tool for the long-term monitoring of common species through the engagement of various volunteers, permitting data acquisition on the long term and over large spatial scales. Despite this substantially increased sampling effort, detectability issues imply that even common species may remain unnoticed at suitable sites. The use of structured CS schemes, including repeated visits, enables to model the detection process, permitting reliable inferences of population size estimates. Here, we relied on a large French structured CS scheme (EPOC-ODF) comprising 27,156 complete checklists over 3,873 sites collected during the 2021–2023 breeding seasons to estimate the population size of 63 common bird species using hierarchical distance sampling (HDS). These population size estimates were compared to the previous expert-based French breeding bird atlas estimations, which did not account for detectability issues. We found that population size estimates from the former French breeding bird atlas were lower than those estimated using HDS for 65% of species. Such a prevalence of lower estimations is likely due to more conservative estimates inferred from semi-quantitative expert-based assessments used for the previous atlas. We also found that species with long-range songs such as the Common Cuckoo (*Cuculus canorus*), Eurasian Hoopoe (*Upupa epops*) or the Eurasian Blackbird (*Turdus merula*) had, in contrast, higher estimated population sizes in the previous atlas than in our HDS models. Our study highlights the need to rely on sound statistical methodology to ensure reliable ecological inferences with adequate uncertainty estimation and advocates for a higher reliance on structured CS in support of long-term biodiversity monitoring.

## Introduction

Worldwide bird populations are nowadays caught in the midst of a global, man-driven erosion of biodiversity caused by the synergistic effects of habitat destruction and fragmentation, resource overexploitation, climate change, pollution, pesticide use as well as the global spread of invasive species (Ceballos et al., 2015; Johnson et al., 2017). In Europe, the intensification of agriculture crystallises this phenomenon because the ever-increased use of pesticides and fertilisers has been pointed out as the main driver of current bird population declines (Rigal et al., 2023). Recent researches have pointed out how important species-specific life attributes (*e.g*., species range and density) and functional traits (*e.g*., body mass, diet or dispersal) explain long-term bird trends and responses to global changes (Hong et al., 2023; Santini et al., 2023; Germain et al., 2023). Monitoring long-term as well as shorter-term trends have been historically possible through standardised biodiversity—including bird—surveys at a national scale and aggregated at the continental one (Jiguet et al., 2012; Pilotto et al., 2020; Brlík et al., 2021). This monitoring produces comprehensive distribution atlases pointing out current ranges and their historical changes (Keller et al., 2020), as well as long-term population trends (Brlík et al., 2021). Identifying common species declines in the long-term should be accounted for in agricultural and planning policies (Gaston & Fuller, 2008; Rigal et al., 2023) to help reconciling society’s needs with the safeguarding of ordinary biodiversity (Couvet & Ducarme, 2014).

Monitoring the success of the implementation of such policies is possible through the measurement of Essential Biodiversity Variables (Jetz et al., 2019; Navarro et al., 2017; Pereira et al., 2013), including the assessment of species distribution range and population abundance or density (Santini et al., 2023). Global monitoring capabilities of species populations have increased over the past few decades as citizen science (CS) has gained prominence across various domains, particularly in ecology (Kullenberg & Kasperowski, 2016). This participatory approach has provided the public with unprecedented opportunities to contribute to biodiversity monitoring through data collection and indirectly through their engagement in policy evaluation (McKinley et al., 2017). The development of CS can be related to the emergence of multiple online databases (Newman et al., 2012) capable of gathering comprehensive datasets across large spatial scales and multiple taxa such as iNaturalist (www.inaturalist.org) and Biolovision (data.biolovision.net) or taxa-centred, for instance, eBird (www.ebird.org). The emergence of CS databases has thus resulted in an exponential increase in biodiversity monitoring capabilities, ranging from phenological shifts (Hurlbert & Liang, 2012), species distribution (Suzuki-Ohno et al., 2017; Matutini et al., 2021) and abundance estimation (Callaghan, Nakagawa & Cornwell, 2021).

Traditionally, species autecology was recensed in atlases providing temporal snapshots of known distribution and population size estimates using discontinuously gathered data collected during a short period (Donald & Fuller, 1998) and allowing long-term changes assessment when repeated over decades (Keller et al., 2020). The first attempt of French avifauna compilation dates back to 1936 (Mayaud, de Balsac & Jouard, 1936) while the first national atlas was published in 1976 (Yeatman et al., 1976) followed by subsequent in 1995 (Yeatman-Berthelot & Jarry, 1995) and 2015 (Issa & Muller, 2015). Each iteration was marked by a significant increase in participating citizen birders, ranging from 500 to 2,400 to 10,000. However, while the ultimate aim of bird atlases is to acquire even knowledge across multiple species for a given geographical area (typically national-wide inquiries), it is essential to note that rare and threatened species benefit from more in-depth population monitoring than more frequent and abundant ones (Ingram et al., 2021). Such exhaustive knowledge of rare species populations is due to interest risen by high extinction risk (IUCN, 2001), the need for recovery plans (Farrier, Whelan & Mooney, 2007) and narrow distributions allowing exhaustive counts (Quaintenne et al., 2020). In contrast, common species (being altogether abundant, widespread; Rabinowitz, 1981) are considered least concern (LC). They consequently tend to receive lesser attention than rarer species (Neeson et al., 2018) despite being key components of global avian biomass changes (Gaston & Fuller, 2008; Inger et al., 2015; Whelan, Şekercioğlu & Wenny, 2015; Rigal et al., 2023).

The principal cause of low quantitative coverage of common birds can be tied to the lack of specific funding stemming from a conservation prioritisation approach (Brooks et al., 2006; Meine, Soulé & Noss, 2006) and the trade-off between data quality and data acquisition over large spatial scales (Devictor, Whittaker & Beltrame, 2010; Kamp et al., 2016). In France, this incomplete knowledge translates into a significant drop in quality for population size estimates of most common bird species, where 60% of breeding species population estimates are qualified as medium (*i.e*., state of knowledge of species abundance considered more or less satisfying, but semi-quantitative data are either lacking or outdated, see Comolet-Tirman et al., 2015) while for rare and localised species (24% of species), estimates are considered as highly reliable (Comolet-Tirman et al., 2022). To account for this discrepancy, a semi-quantitative estimation method was used for the last atlas survey, with population sizes simply inferred from the average number of breeding pairs measured over 10 × 10 km grid cells using abundance classes of 1–9, 10–99, 100–999, 1,000–6,666 (the last upper limit could vary according to knowledge about species densities) multiplied by the number of grid cells known for nesting. Population sizes estimated using this method correspond to lower and upper limits. Lower limits were obtained by calculating the geometric mean of the abundance classes weighted by the number of 10 × 10 grid cells belonging to these abundance classes while upper limits were obtained by using the arithmetic means (see Roché, Muller & Siblet, 2013 method referred further as *ArGeom*).

However, similarly to other studies (Kellner & Swihart, 2014), this approach fails to account for species detectability *p* defined as the probability of detecting at least one individual of a given species in a particular sampling effort, given that individuals of that species are present in the area of interest during the sampling session (Boulinier et al., 1998). Numerous studies have previously shown that p varies with time of day and season (Skirvin, 1981), observers (Quinn et al., 2011) and year-specific factors (Kéry & Schmid, 2004). Omitting species detectability by assuming perfect or constant p across sampling schemes, observers and habitat types can lead to biased inferences (Nichols, Thomas & Conn, 2009; Kéry, 2011) and affect the estimation of long-term trends due to its unaddressed variation (Schmidt, McIntyre & MacCluskie, 2013; Sanz-Pérez et al., 2020).

Here, we propose an estimation method enabling a more robust approach of population size estimations. We provide associated uncertainty intervals built upon a revised structured sampling scheme, ensuring data traceability and allowing inferences in the spatial variation of species abundance by formally including the detection process within the modelling framework.

In this study, we aimed at testing whether applying this modelling framework on an unprecedentedly large citizen-based dataset collected over France would (i) provide a new quantitative evaluation of French breeding bird populations and (ii) allow a comparison of population sizes inferred through Hierarchical Distance Sampling (HDS) from those inferred using the previous atlas methodology *ArGeom* across a large part of French avifauna. In particular, we expect that quantifying the influence of species detectability would allow more relevant ecological inferences (*e.g*., including environmental and sampling effort covariates to the models) to approach closer to a realistic estimation of breeding bird population size at a national level than previously used methodologies. A preprint version of this article has been peer-reviewed and recommended by PCIEcology (https://doi.org/10.24072/pci.ecology.100683).

## Materials and Methods

### Sampling protocol

EPOC-ODF (French structured estimation of breeding bird population size) is a French CS monitoring scheme based upon 5-min point counts, where observers are tasked to point locations of recorded individuals, either through visual or auditory detection. Birders can register their field observations directly using the NaturaList smartphone application or transcript later on the data portal Faune-France (www.faune-france.org). The survey locations corresponded to the centroids of a 2 × 2 km grid, selected from a random sampling. Each location has to be visited three times during the breeding season, from March to June, each consisting of three successive 5-min point counts, to limit chances of duplicated counts while being less demanding in observation effort (Fuller & Langslow, 1984). After completion, *i.e*., nine visits during a breeding season, surveyed sites are removed from the sampling pool for the subsequent year, to maximise the number of sites surveyed. See Materials S1 for more details about the sampling design.

**Figure 1:**
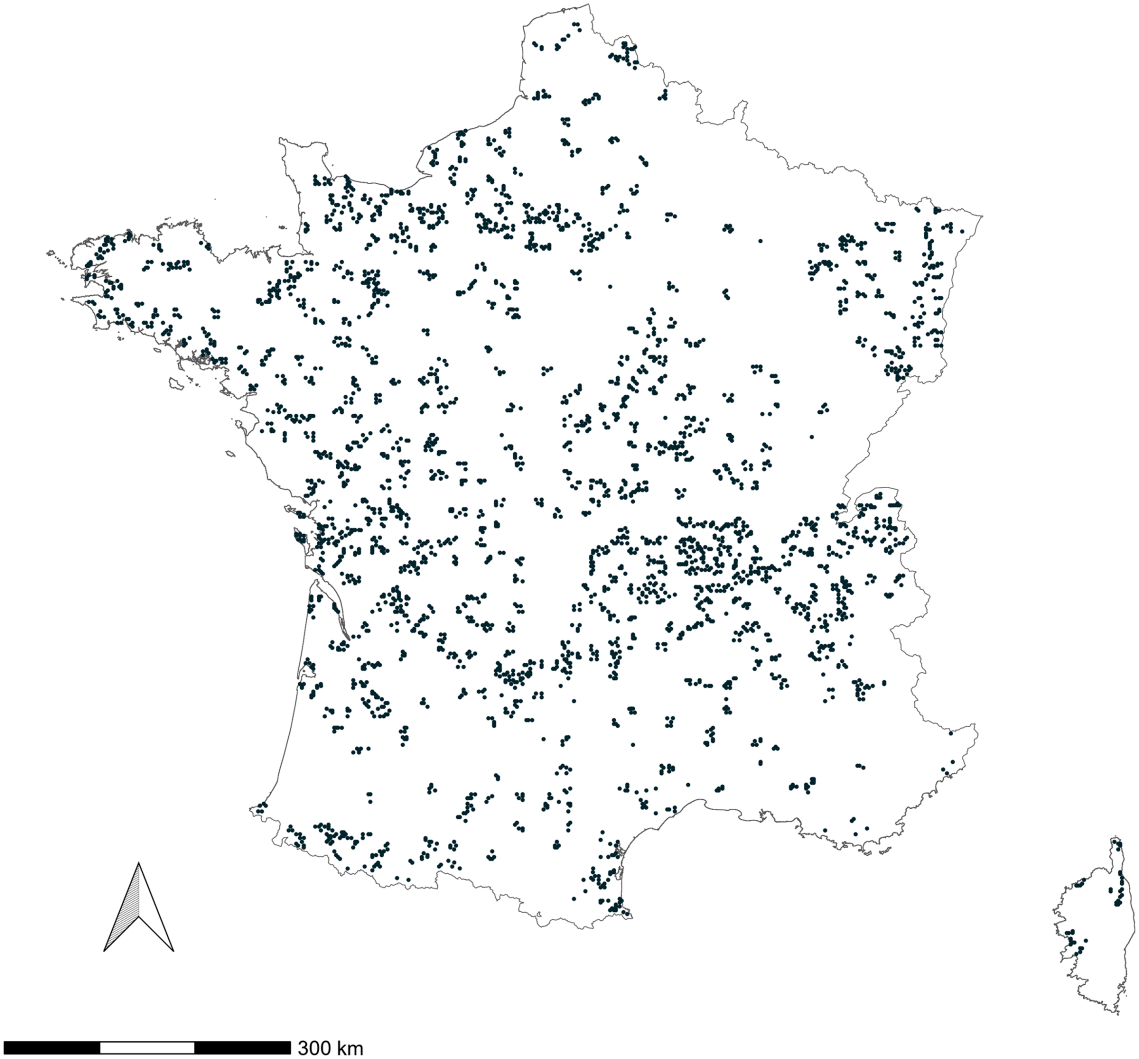
Spatial distribution of surveyed EPOC-ODF locations (*n* = 3,505) over 2021–2023 breeding seasons.

Over the 2021 and 2023 breeding seasons, 276 distinct species were encountered over 27,156 complete checklists collected over 3,873 pre-selected locations (Fig. 1) by 520 observers. Sampling effort is monitored through local associations tasked to recruit volunteers. The primary focus of the scheme being the monitoring of common breeding bird species, we decided to constrain the number of species considered viable targets of this scheme to 103 out of the 276 species contacted. We narrowed our study to 63 species out of the initial set of 103, comprising only those recorded at a minimum of 150 distinct locations (3.9% of total locations), to have a sample size allowing to reach model convergence. We also applied a temporal filter that considered both observed activity during the breeding season and expert opinion to capture the breeding phenology of each targeted species and exclude possible early or late migrants from population size estimates (see Materials S2.1).

### PCA reduction of environmental covariates

For bioclimatic data, we used 19 variables from WorldClim at 1 km resolution (Fick & Hijmans, 2017), on which we applied a Principal Component Analysis (PCA), keeping the first three axes (82.3% of explained variance), to limit multicollinearity through orthogonal transformation (Cruz-Cárdenas et al., 2014).

**Figure 2:**
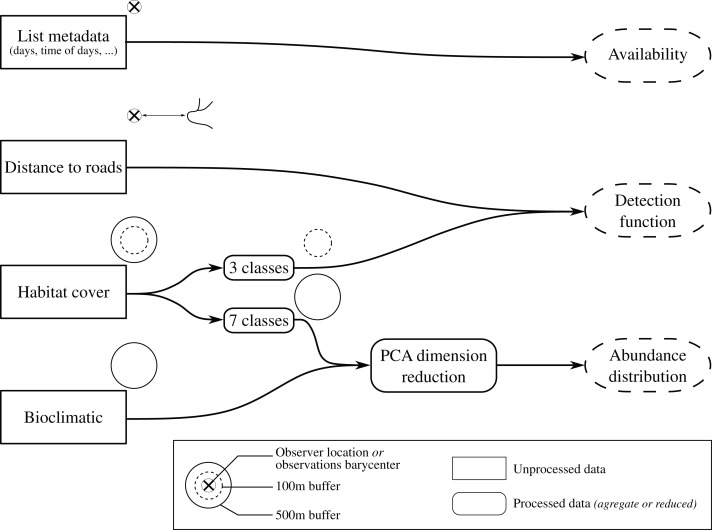
Global overview of covariates acquisition, treatments and usage workflow. Data are retrieved over observers’ GPS location or approximated using observations barycenter, when unavailable, over two resolutions, 100 m buffer (dotted circles) and 500 m buffer radii (solid circles). Distance to roads is determined by measuring the distance between the nearest road to the observer location or observation barycenter. Habitat cover, in percentage, is aggregate over seven and three classes (see Table S3.1). Seven-class habitat cover and bioclimatic are reduced from PCA keeping the first three dimensions for bioclimatic data and three selected for the seven-class habitat cover.

We used habitat cover data from Theia OSO at 10 m resolution (Thierion, Vincent & Valero, 2022) and aggregated it according to two different scales: (1) a seven-class corresponding to habitat type (Urban, Annual crops, Perennial crops, Pastures, Grasslands, Forests, Water body/Mineral surfaces) and (2) three-class (Open, Forests, and Artificial) in regards to overall effect on detectability (Fig. 2). Additionally, we conducted PCA dimension reduction on the seven-class aggregation, retaining three of the six PCA (54.71% of explained variance) axes depicting environmental gradients for (i) forest-to-open-field cultures; (ii) open-field cultures-to-pastures and (iii) perennial crop-to-urban Materials S3 for the workflow pipeline and habitat cover aggregation. Distances to roads were measured from ROUTE 500 (Cote et al., 2021). Environmental covariates were extracted over a 500 m buffer radii upon registered observer location (Fig. 2). These distances were chosen according to mean dispersal distances and home range sizes in common European birds (Paradis et al., 1998). The three-class habitat covers were collected upon 100 m circles radii to assess immediate habitat types that could hinder species detection. Whenever the exact location was unavailable, we used the centroid of sightings as a proxy for observer location (Materials S4). We used environmental data collected from a prediction grid covering France at a resolution of 2 × 2 km for PCA dimension reduction. Outcomes from this initial PCA were used to transform environmental data collected from surveyed locations through PCA projections.

### Modelling framework

We used Hierarchical Distance Sampling (HDS) models to estimate the abundance of the target species while accounting for uncertainty arising from the observation process (Chandler, Royle & King, 2011; Kéry & Royle, 2015). We applied a right-side truncation of 5%, removing observation distances above the 95% quantile, for each targeted species to remove extreme distance values for model robustness (Buckland et al., 2001). Then, we divided observation distances into five proportional bin classes based on the maximal observed distance. Models calibration and assessment were done using unmarked 1.2.5 R package (Fiske & Chandler, 2011). Effort covariates were accounted for by incorporating the Julian date and the hours of list realisation (as minutes from sunrise), see Table 1.

**Table 1:** Ensemble of sub-models tested in the secondary candidate set approach (Morin et al., 2020).

States	Sub-models
Detection	~ Distance to roads
	~ Distance to roads + Proportion of artificial lands (100 m)
	~ Distance to roads + Proportion of open lands (100 m)
	~ Distance to roads + Proportion of forests (100 m)
Availability	~ Julian date
	~ Julian date + Julian date²
	~ Hour (*)
	~ Hour + Hour²
	~ Julian date + Hour
	~ Julian date + Julian date² + Hour
	~ Julian date + Hour + Hour²
	~ Julian date + Julian date² + Hour + Hour²
Abundance	~ 3 Bioclimatics PCA axis + 3 Habitat cover PCA axis

**Note:**

(*) For the hour effort covariate, we used minutes from sunrise estimated from site longitude, latitude and date of list completion.

Distance sampling key functions, depicting detection probabilities fall-off given distance of observation (Buckland et al., 2001), were chosen between half-normal and hazard-rate based on Akaike Information Criterion (AIC, Akaike, 1974), with other states kept constant.

**Figure 3:**
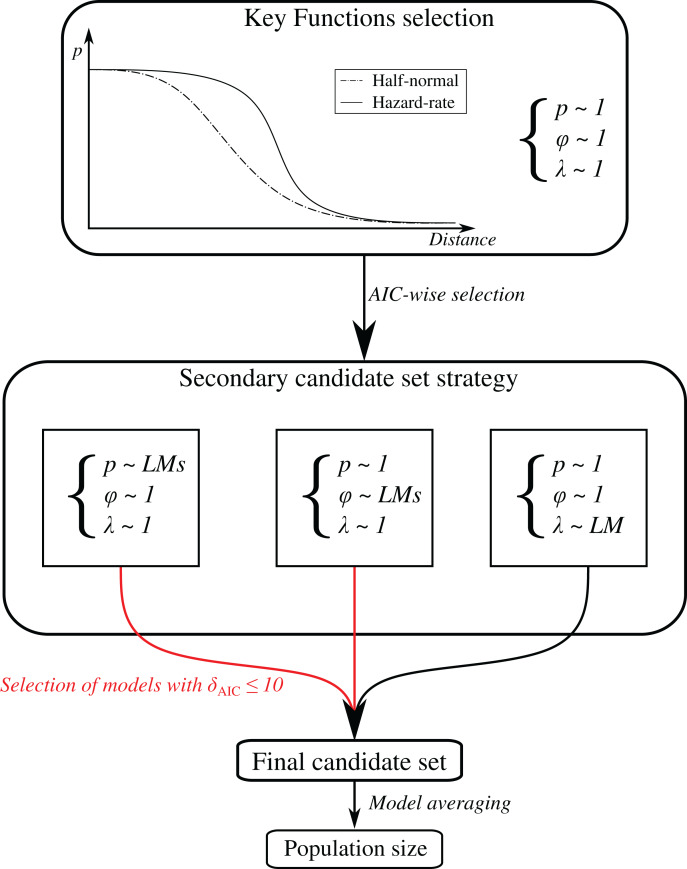
Methodological framework for population size estimation. At first, a key function is determined AIC-wise between half-normal and hazard-rate with other components of the HDS kept constant. The selected key function is then used during the secondary candidate set strategy (Morin et al., 2020), fitting multiple sub-models (Table 1) for each state separately holding others constant. Sub-models with greatest support (*δ_AIC_* ≤ 10) are then selected in a final candidate set consisting of multiple HDS through combinatorial association (Morin et al., 2020). At this stage, we used sub-models estimated coefficients as starting values to help model convergence. Population size estimates are obtained through model averaging of the final candidate set. *LM: Linear Model*.

We based our modelling framework on a secondary candidate set strategy (Fig. 3), where the detection and availability states of our HDS were fit according to the set of the first candidates while others were kept constant (Morin et al., 2020). For the Poisson process underlying abundance distribution, we used a single model consisting of retained covariate PCAs axes (Table 1). See Table S5.1 for the number of times where each sub-process was included in the final candidate sets.

HDS population size estimates were obtained by averaging retained secondary candidate sets models, based on their relative model performance using AICc (Fig. 3). We excluded the Eurasian Sparrowhawk (*Accipiter nisus*), the Meadow Pipit (*Anthus pratensis*) and the Coal tit (*Periparus ater*), from model averaging and exclusively relied upon prediction from best final models owing to substantial differences observed among their secondary candidate sets models.

Model goodness-of-fit was assessed using an oveardispersion coefficient metric ( $\hat C$; Johnson, Laake & Ver Hoef, 2010). We used the chi-square metric as the discrepancy measure between observed and expected counts. Computed $\hat C$ corresponds to the ratio between the chi-square obtained from the fitted model to the mean of bootstrapped chi-squares obtained from simulated datasets based upon estimated parameters (Kéry & Royle, 2015). All models were fit according to a Poisson (P) distribution after top model assessment and calculation of $\hat C$, secondary candidate sets with $\hat C$ top models exceeding 1.2 were calibrated using a negative binomial (NB) distribution (Payne et al., 2018). For a global overview of our modelling approach, see Materials S5. Out of 63 species, we excluded nine species from the analysis; three exhibited signs of underdispersion with $\hat C$ values less than 0.9 while six had $\hat C$ values exceeding 1.5 (Payne et al., 2018), showing signs of overdispersion, despite being calibrated using a negative binomial distribution, see Table S2.1 for more details.

We assessed the robustness of our estimations to the exclusion of one year of data, corresponding to a third of the global dataset. We compared population size estimates from EPOC-ODF data collected over 2021–2023 to estimates obtained from EPOC-ODF data collected over 2021–2022. Using the 2021–2022 subset, we estimated the population sizes of 30 species, detected in at least 150 distinct sites. From these 30 species, seven mean population sizes estimated using 2021 to 2023 data were outside the confidence intervals estimated from 2021 to 2022 data, with a slightly smaller population size estimated (Table S2.2) overall highlighting robust estimations.

### Trimming of HDS population size estimate: assessment of model extrapolation

Population sizes were obtained by summing predicted values over the prediction grid. As we intend to predict over a large surface, novel environmental conditions may arise, leading to possible dissimilarities between the environmental gradient collected at survey sites and the environmental gradient over novel conditions (Elith & Leathwick, 2009). Mesgaran, Cousens & Webber (2014) described two types of extrapolation; (i) novelty type I (NT1) where projected points (*i.e*., prediction grid) are outside the range of individual covariates collected by the sampling scheme and (ii) novelty type II (NT2) depicting the case when projected points are within univariate range but constitute novel combinations between covariates.

**Figure 4:**
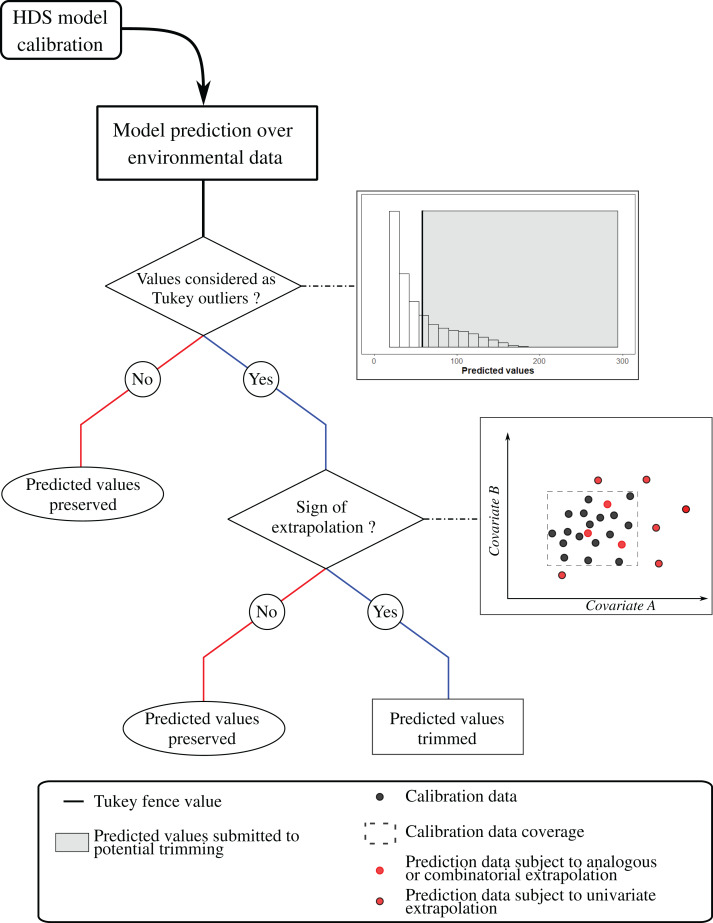
Decision tree of the post-prediction treatment. First, we analysed the distribution of predicted abundance values across the prediction grid and detected outliers, using a hinge of k = 1.5 (Tukey, 1977). We compared each environmental condition of the prediction grid cell with the environmental condition collected by the sampling scheme and used for model calibration. When a prediction cell depicted signs of NT1 extrapolation and its estimated abundance was considered as an outlier, we trimmed the predicted grid cell abundance to the Tukey fences value.

We trimmed predicted values (Fig. 4) over prediction grid cells showing signs of NT1 extrapolation, to a threshold value determined using a Tukey fence (Tukey, 1977), estimated from the distribution of predicted values with k = 1.5. Extrapolation assessments were done using *dsmextra* 1.1.5 R package (Bouchet et al., 2020). We used this post-prediction treatment to assess population size estimates stability. We measured the coefficient of variation, corresponding to the ratio between the standard deviation and the mean of the “untrimmed” and “outlier-trimmed” estimated range uncertainty. Large coefficients of variation imply great discrepancies in confidence intervals of untrimmed and outlier-trimmed estimated uncertainty intervals. This is mainly caused by the spatial filtering from the extrapolation assessment highlighting smaller geographic regions with similar environmental conditions from sampled ones and the trimming of predicted abundance outliers. Species with a coefficient of variation exceeding 30% were removed from the comparison of *ArGeom* and HDS population size estimates (Table S2.3).

### Comparison of *ArGeom* and HDS estimated population sizes

For comparable estimates between *ArGeom* and HDS approaches, we restricted the prediction grid area species-wise for HDS estimation according to the distribution of their known breeding locations, collected over a 10 × 10 km grid during the previous French atlas (Issa & Muller, 2015). To estimate breeding populations of species for which male identification was possible, either male vocalisations or visual distinctions because of sexual dimorphism, an ad-hoc filter was applied (Table S2.1), resulting in HDS estimates reflecting the male counts for those species.

**Figure 5:**
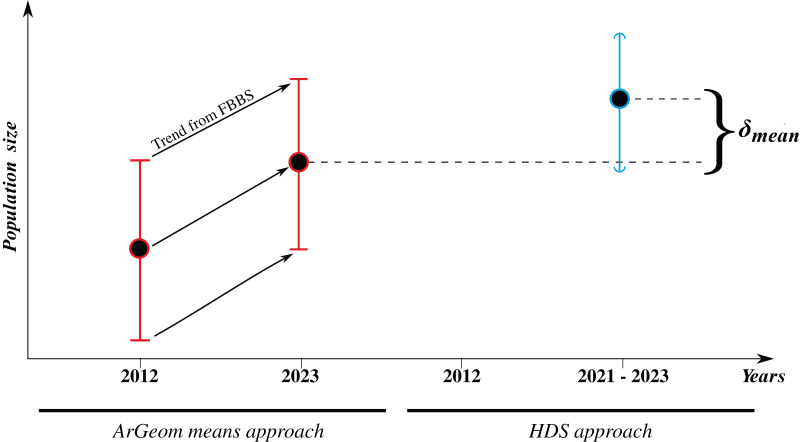
Population size estimates comparison methodology, *ArGeom* in red and HDS in blue. *ArGeom* estimates for 2012 were obtained through calculations using both arithmetic and geometric means (Roché, Muller & Siblet, 2013) and were then updated to 2023 using specific mean trend estimates from FBBS (Table S2.1). Flat intervals signify min and max value estimates, while curved intervals signify confidence intervals.

As the *ArGeom* approach estimated species bird population sizes as a number of breeding pairs (Roché, Muller & Siblet, 2013), for species where male identification in the field was impossible (no sexual dimorphism), we used all available data, after applying the phenological filter, and divided HDS estimates by two for comparable estimates with *ArGeom* population sizes. After retrieval of *ArGeom* estimates from the previous atlas (Issa & Muller, 2015), we updated these estimates using recent population trend estimates derived from the French breeding bird survey (FBBS; Jiguet et al., 2012) data spanning 2012–2023 (Table S2.1). Given the absence of a mean estimate in the *ArGeom* approach, we approximated it using the midpoint between the maximum and minimum estimated (Fig. 5).

To study the differences between the two approaches, we measured ${\delta _{mean}}$ corresponding to the percentage of the difference between HDS and *ArGeom* estimates.

$${\delta _{mean}} = \; \displaystyle{{\left( {Estimate{s_{ArGeom}} - Estimate{s_{HDS}}} \right)} \over {\left( {Estimate{s_{ArGeom}} + Estimate{s_{HDS}}} \right)/2}}.$$

### Study of variation of estimated population sizes between the two approaches

As species detectability stems from physical traits and vocalisations, phylogenetic related species tend to have the same detectability (Johnston et al., 2014; Sólymos et al., 2018). We calibrated a Phylogenetic Generalised Linear Mixed Model (PGLMM) using the phyloglmm 1.0 (Li & Bolker, 2019) R package. We study ${\delta _{mean}}$ variations across species while implementing a random effect covariance structured based on phylogenetic relatedness using phylogenetic distances retrieved from Burleigh, Kimball & Braun (2015). The PGLMM model was calibrated using (i) extracted detection probabilities from the availability state estimated through HDS (Fig. 3 and Materials S5) after model averaging of the final candidate sets models in regards to AICc scores, and (ii) *ArGeom* uncertainty as fixed variables. For *ArGeom* uncertainty, corresponding to the difference between maximal and minimal estimated values, we relied on the decimal logarithm to limit variation in ${\delta _{mean}}$ solely due to different population size magnitudes.

Response weights consisted of normalised weights from the inverse of uncertainty around FBBS trends between 2012 and 2023 (Table S2.1), divided by the mean to limit excessive weight attribution and facilitate model convergence.

## Results

### Species trends over 2012–2023

From 2012 to 2023, out of 63 bird species, 15 showed a significant decrease ($\bar x$ = −22.79% $\pm$ 14.84) in total population size, while 16 showed a significant increase ($\bar x$ = 28.02% $\pm$ 22.52; see Materials S2 for species-related FBBS trends).

### HDS population size estimations

Out of the 54 species with acceptable values of overdispersion ( $\hat C$) using the *HDS* approach, we excluded eight species showing large discrepancies in population size estimates (Materials S2, Table S2.3) between pre- and post-prediction treatment (Fig. 2.).

Out of the remaining 46 species used for comparison between *ArGeom* and *HDS* estimates, *HDS* models showed acceptable values of overdispersion ( $\hat C$) ranging from 0.94 to 1.2 ( $\bar x$ = 1.07 $\pm$ 0.06) for 38 species calibrated using a Poisson distribution and 1.09 to 1.47 ( $\bar x$ = 1.27 $\pm$ 0.13) for eight species calibrated using a Negative binomial distribution.

### Population size comparison between *ArGeom* and HDS

Across all species, estimated mean density ranges from 0.09 to 27.51 individuals per square kilometre, while ArGeom range uncertainty varies from 3.9 to 6.69 on the decimal logarithm scale corresponding to variations from 7,920 to 4,850,000 in estimated number of pairs. See Tables S6.1–S6.3 for more details about species estimated population size according to *ArGeom* and HDS approaches.

**Figure 6:**
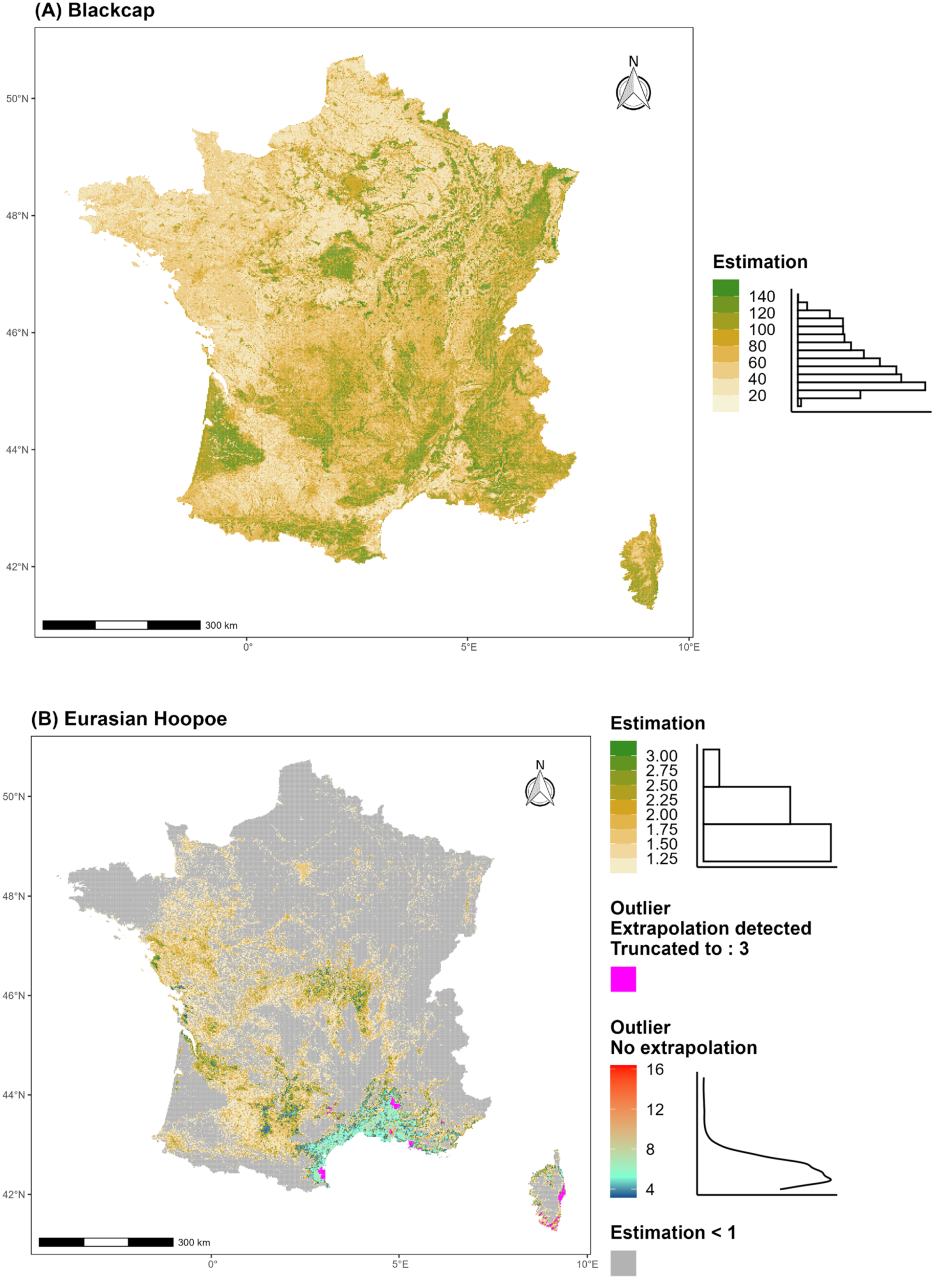
Examples of resulting abundance maps. (A) Blackcap (*Sylvia atricapilla)*, and (B) Eurasian Hoopoe (*Upupa epops)*. Estimations correspond to the number of male, or potential pairs (see Table S2.3) over a 4 km-squared area. Grid cell predictions are categorised into three groups: (1) those with estimated abundance not considered as outliers depicted with a green colour step gradient and its distribution histogram; (2) those with estimated abundance considered as outliers and not subject to NT1 extrapolation, displayed in a blue-to-red gradient, along with their distribution density; and (3) those with estimated abundance considered as outliers with novel environmental conditions subject to NT1 extrapolation highlighted in pink with the Tukey value used for trimming in the post-prediction treatment.

A comparison between updated *ArGeom* and HDS estimated population sizes showed that HDS estimates were higher than *ArGeom* for 30 of the 46 species tested (Table S6.1). Our results suggest lower estimates from *ArGeom* ( ${\delta _{mean}}$ < −0.2), either for open habitat specialists such as European Stonechat (*Saxicola rubicola*), European Goldfinch (*Carduelis carduelis*) or Eurasian Linnet (*Carduelis cannabina*) than for forest generalists such as Great Spotted Woodpecker (*Dendrocopos major*), Blue Tit (*Cyanistes caeruleus*) or Eurasian Blackcap (Fig. 6A) with ${\delta _{mean}}\;$= −0.629 $\pm$ 0.4, over 22 species. Species whose estimations were similar ( ${\delta _{mean}}$ ∈ [−0.2;0.2], with on average ${\delta _{mean}}$ = −0.006 $\pm$ 0.096, over 17 species) between *ArGeom* and HDS included species such as Eurasian Wren (*Troglodytes troglodytes*), Great Tit (*Parus major*), European Robin (*Erithacus rubecula*) and European Nuthatch (*Sitta europea*). Fewer species, mainly characterised by greater maximal observation distances, such as Common Cuckoo (*Cuculus canorus*), Eurasian Hoopoe (Fig. 6B) and European Blackbird (*Turdus merula*) had higher population sizes estimated by *ArGeom* approach compared to HDS ( ${\delta _{mean}}$ > 0.2, with on average ${\delta _{mean}}\;$ = 0.41 $\pm$ 0.148, over seven species; see Materials S6 for population size comparison table).

**Figure 7:**
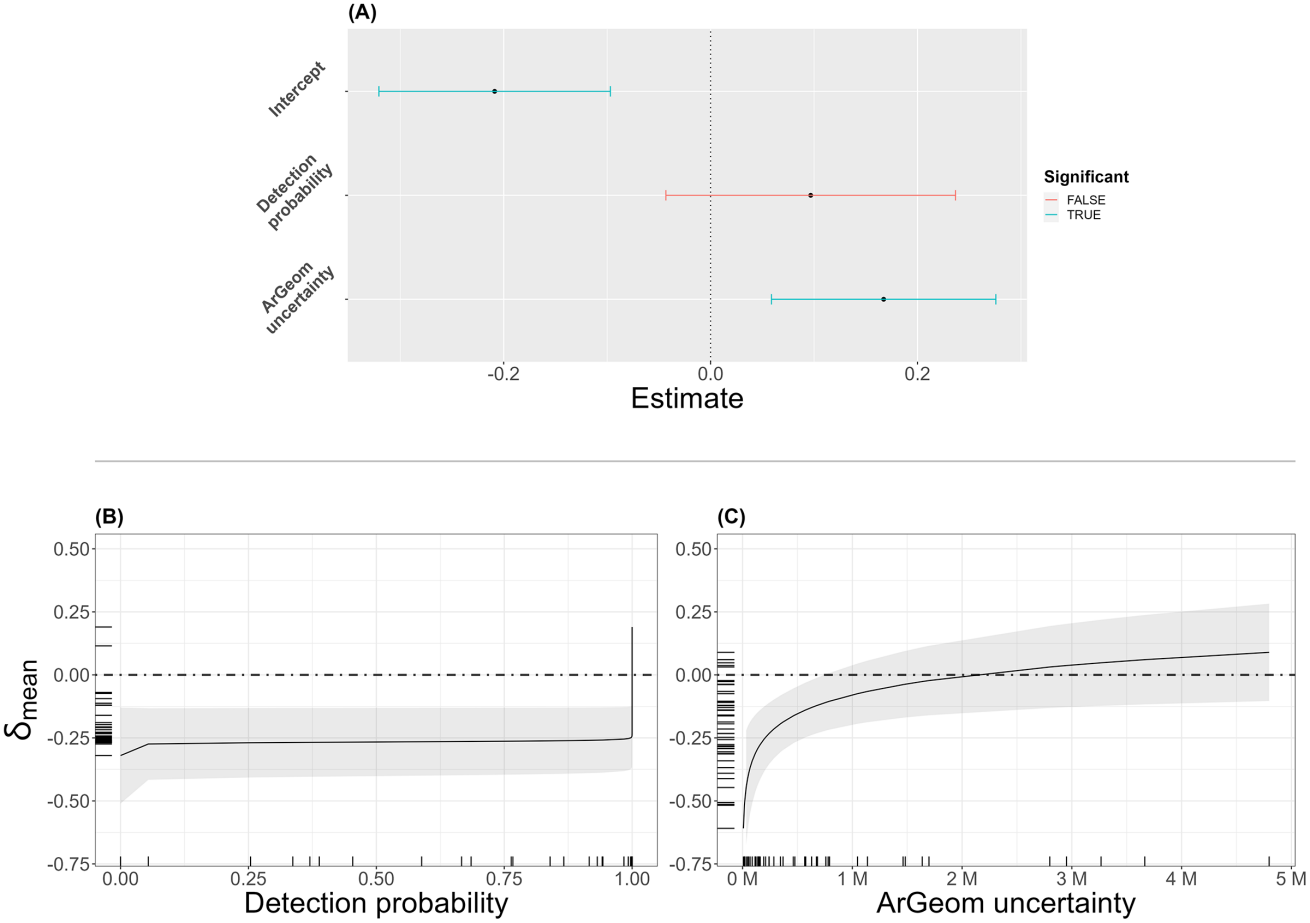
Results from the PGLMM. Confidence intervals of the model coefficient, coefficients significantly different from 0 are represented in blue. Marginal effect plots of population size estimate differences ( ${\delta _{mean}}$) responses. ${\delta _{mean}}$ responses are predicted over gradients of focal terms, either species detection probabilities (B) or *ArGeom* reported uncertainties (C), while other covariates are held constant at their mean. Species detection probabilities and *ArGeom* uncertainty are represented on their natural scales, after inverse logit and inverse decimal logarithm transformation, respectively. Dot-dash line corresponds to a ${\delta _{mean}}$ of 0, signifying estimated population size convergence by the two approaches, negative and positive values of ${\delta _{mean}}$ reflect lower and higher population size estimates of *ArGeom* relative to those obtained using HDS.

Results from the PGLMM (Fig. 7A) showed an overall significantly lower *ArGeom* population size estimates (−0.209, with 95% CI [−0.321 to −0.097], Pval = 0.001), as well as a significant positive effect of *ArGeom* range intervals (0.167, with 95% CI [0.059–0.276], Pval = 0.003) on the differences between the two approaches. Species detection probabilities had no significant effect (0.097, with 95% CI [−0.043 to 0.237], Pval = 0.176) on ${\delta _{mean}}$ variation.

Marginal effect plots from the PGLMM model showed that the mean response of ${\delta _{mean}}$ over species detection probability was predominantly negative, ranging from −0.45 to −0.1 (Fig. 7B), for *ArGeom* uncertainty. This showed that ${\delta _{mean}}$ tended towards the convergence of population size estimates (Fig. 7C) for species with larger estimated interval ranges. There were no signs of multicollinearity (VIF < 5; James et al., 2013) between the two variables.

## Discussion

Our results showed that bird population size estimates from the previous *ArGeom* approach, not accounting for the observation process nor habitat affinity covariates, are predominantly lower than population sizes estimated from the HDS approach, up to 65% of species. While we found that the prior estimated uncertainty ranges from *ArGeom* had a positive effect on the convergence of population sizes estimated by the two methodological approaches (expert estimates based on atlas data *vs* predicted estimates from modelled citizen science data accounting for detection probabilities), we did not find a significant effect of species detection probabilities which could explain the differences between the two approaches. We show that *ArGeom* produces population sizes that are largely lower than those obtained by HDS ( ${\delta _{mean}}$ < 0.2) regardless of habitat specialisation or affinity. This is likely due to the methodology used for *ArGeom* that did not account for the detection process nor for species-habitat relationships when extrapolating locally known abundances to unsampled locations.

Despite the lack of significant evidence for the effect of species detection probabilities on population size estimations, our results tend to corroborate previous studies where ignored detection processes had likely biassed ecological inferences, including species distribution models (Kéry, Gardner & Monnerat, 2010), population trends (Norvell, Howe & Parrish, 2003; Schmidt, McIntyre & MacCluskie, 2013) and population sizes (Kéry, Royle & Schmid, 2005). This lack may be especially true in a context of global change, where avian breeding phenology showed evidence of shifts towards earlier breeding over the years (Parmesan, 2007; Devictor et al., 2012; Gaüzère & Devictor, 2021) to synchronise with their food sources (Visser, Holleman & Gienapp, 2006; Michel et al., 2016). Such shifts induce diverse species-related seasonal and inter-annual changes in detectability that need to be accounted for, particularly for schemes spanning over multiple species (Lehikoinen, 2013).

In France, the *ArGeom* approach was developed in the context of moderate semi-quantitative data collection with an acknowledged uneven participation across the territory (Roché, Muller & Siblet, 2013; Issa & Muller, 2015). The semi-quantitative data collection was based on the estimation of the number of breeding pairs over 10 × 10 km grids derived from a mixture of count prospects and expert opinions collected over 1,953 out of 5,879 10 × 10 km grids (Issa & Muller, 2015). As the primary goal of this approach was to give a likely magnitude of population size across the territory (Roché, Muller & Siblet, 2013), *ArGeom* intervals were produced by the extrapolation from the initially prospected 10 × 10 km grids to all metropolitan grid cells considered suitable for breeding, after the detection of potential and confirmed breeding evidence. Although proved useful and relevant to assess population sizes when large-scale quantitative data on species occurrences are lacking or are unevenly distributed, such a methodology implies greater uncertainty intervals for abundant and broadly distributed species and smaller intervals for scarce and narrowly distributed species when not accounting for measurement uncertainty.

Contrary to generalist species, which have widespread distributions due to broader habitat niche breadths, specialists are generally more localised (Clavel, Julliard & Devictor, 2011) and typically use a smaller range of habitats (Julliard et al., 2006). Despite a growing interest in rare species-focused monitoring (Fontaine et al., 2022), citizen sciences programs are mainly designed for large-scale multi-species surveys (Devictor, Whittaker & Beltrame, 2010). Citizen science schemes balance between a trade-off among data quantity and data quality, corresponding to either the acquisition of a great quantity of unstructured scheme, or the acquisition of standardised data implying replicated visits over randomly sampled locations (Devictor, Whittaker & Beltrame, 2010). As such, in the first case, citizen science schemes could be more prone to false-negative errors, resulting in biassed inferences over habitat cover relations due to omission of the detection process (Johnston, Matechou & Dennis, 2022). In the second case, given the small habitat range of specialist species and the scale of the territory sampled (*e.g*., here the metropolitan French territory), citizen science sampling schemes could be representative of the entire territory sampled but with a higher risk of missing some key habitats and associated specialist species.

### Potential consequences for community-level assessments

A recent study about long-term effects of climate and land use changes on bird communities (Gaüzère et al., 2020) showed that both generalist cold-dwelling species, such as the Common Chiffchaff (*Phylloscopus collybita*) or the Eurasian Blackcap, and warm-dwelling species, such as the Common Nightingale (*Luscinia megarhynchos*) had the most substantial negative and positive contributions to the trend in Community Thermal Index (CTI), a community-weighted index representing the realised thermal niche of a community based upon species relative abundance and species thermal indices (STI). In the present study, these species tended to have lower population sizes estimated when the detection process was omitted compared to estimates based on our modelling approach. As a result, this could affect the estimations of their contribution to the calculation of community-weighted mean indices, such as CTI, and therefore bias the estimation of the trend in community thermal response and subsequent studies of aggregated indices, which are known to display large regional variation (Rigal & Knape, 2024). We, therefore, suggest that considering the detection process in studies relying on community-weighted indexes by species’ relative abundances could be as important as it is for estimating population sizes.

Community indices such as species diversity (Ricotta, 2005) and functional diversity (Villéger, Mason & Mouillot, 2008; Gaüzère et al., 2019) are commonly use species relative abundance as a basis, without taking into account the detection process (Pillar & Duarte, 2010), despite multiple studies showing it could affect community indices inference (Tingley & Beissinger, 2013; McNew & Handel, 2015; Jarzyna & Jetz, 2016; Richter et al., 2021).

### Conservation implications

Our study also suggested that lower or higher population sizes estimated from *ArGeom* were not randomly distributed among species according to their conservation status. Out of the 46 species estimations used in the comparison analysis, 10 had an unfavourable conservation status in France (*i.e*., lower than Least Concern, LC; UICN France et al., 2016).

Among these species of conservation concern, two species, European Greenfinch (*Carduelis chloris*) and European Turtle Dove (*Streptoptelia turtur*), showed no signs of difference in their population sizes. By contrast, five species, European Stonechat, Barn Swallow (*Hirundo rustica*), Red-backed Shrike (*Lanius collurio*), Eurasian Kestrel (*Falco tinnunculus*) and Willow Warbler (*Phylloscopus trochillus*) considered as NT (Near Threatened) and three species, Eurasian Linnet, European Goldfinch and European Serin (*Serinus serinus*) considered as VU (Vulnerable) had lower population sizes estimated from *ArGeom* than from HDS approach (NT: ${\bar \delta _{mean}}\;$ = −0.608 $\pm$ 0.217 and VU: ${\bar \delta _{mean}}\;$ = −0.667 $\pm$ 0.146). Our results showed that these species may need a reevaluation of their conservation status and highlight the need to rely on hierarchical models taking account of the detection process in ecological inferences, given that potential misclassification of population conservation status may arise from process noise and observation error (Connors et al., 2014). As conservation policy decisions depend on uncertainty levels (Williams, 2003; Freckleton, 2020), assessing measurement error through the integration of the detection process (Nichols et al., 2011) could provide more reliable ecological inferences (Guillera-Arroita et al., 2014). CS schemes are becoming more and more a reliable source of data to ensure biodiversity monitoring (Chandler et al., 2017) and can, through standardisation (Buckland & Johnston, 2017; Johnston et al., 2019), contribute to the calibration of data-hungry models such as hierarchical models for reliable ecological inferences (Isaac et al., 2020; Kéry & Royle, 2021; Johnston, Matechou & Dennis, 2022).

### Comparison to other European countries

Another way to assess the relevance of the two estimation approaches would be to compare their population size estimates to the ones obtained from other European countries, using a ratio between countries to produce comparable estimates. Such an approach should however be used with caution because it would be limited by comparability in habitat repartitions or biogeographical considerations among different European countries. To go further into inter-country comparisons, we relied on the German population sizes estimated for the previous European Bird Directive (BirdLife International, 2021) obtained from both point count and territory mapping methods (Gedeon et al., 2015). For abundant species such as the Blackcap ( ${\delta _{mean}}$ = −0.29; German population size expressed in millions of pairs = [7.17–9.49]), both approaches led to similar results than German population estimates, while HDS estimates were closer to German population sizes for the Firecrest (*Regulus ignicapilla*; ${\delta _{mean}}$ = −0.46; [1.92–2.85]) and the Blue Tit ( ${\delta _{mean}}$ = −0.42; [5.01–7.41]). For species with higher population sizes estimated by *ArGeom* than HDS ( ${\delta _{mean}}$ > 0.2), the Common Cuckoo ( ${\delta _{mean}}$ = 0.25; [0.58–0.95]) and the Corn Bunting (*Emberiza calandra*; ${\delta _{mean}}$ = 0.6; [0.25–0.44]) showed estimates of German populations closer to the HDS than the *ArGeom* approach. Finally, for the Common Whitethroat (*Curruca communis*; ${\delta _{mean}}$ = 0.42; [0.93–1.47]), the German population size is closer to *ArGeom* estimates (see Materials S6.4 for additional information).

Regarding magnitudes, both approaches produced similar estimates compared to German ones. However, due to different sampling and modelling methods used, these formal comparisons, although informative, need to be more fully satisfying and highlight the discrepancies in sampling and analytical methods across the European continent (Keller et al., 2020). Such differences could be accounted for, either by (i) a global standardisation of schemes, as promoted by the PECBMS (Pan-European Common Bird Monitoring Scheme; Brlík et al., 2021) for species trends, but also (ii) through the use of Integrated Models (IM) capable of mobilising data from multiple and somewhat heterogeneous sources (Isaac et al., 2020; Zipkin et al., 2021).

### Study limitations

Our approach relies on data collected from the EPOC-ODF structured CS schemes, providing data with repeated visits. However, as is, the frequentist framework of *unmarked* R package (Kellner et al., 2023) does not permit inferences on social species occurring in large flocks. Taking account of social species during the breeding season (corresponding to 1/10th of the scheme targeted species) would therefore require a Bayesian framework to include the effect of flock size on species detectability (Clement, Converse & Royle, 2017).

Given the timeframe and the sampling design, *i.e*., all sites are not visited every year to maximise the number of total surveyed locations, it is not possible to estimate species demographic parameters, such as survival and recruitment (Sollmann et al., 2015; Schmidt & Rattenbury, 2018). We also assumed a sex ratio of 1:1 for species without sexual dimorphism, during the breeding season, which could potentially bias estimates for species deviating from this assumption. Taking account of species population structure requires frameworks such as Integrated Population Models (IPM; Schaub & Ullrich, 2021) and specific data collection (King, 2014), for instance, bagging or nest surveillance.

As obtaining relevant predictions of species abundance over unsampled environmental conditions was one of our main methodological challenges, we used environmental data condensing habitat information (Tredennick et al., 2021). To fit our statistical framework, we assumed that most bird species would interact with their habitat following a linear relationship (see Fig. 3). We therefore used PCA reduction to summarise species linear responses to national-scale habitat gradients including forest-to-open-field cultures, open-field cultures-to-pastures and perennial crop-to-urban habitats (see Materials S3). PCA reduction permits model convergence by condensing complex habitat structures to a small number of environmental covariates, though it could bias estimates of species thriving in a specific habitat restricted to the extreme edge of the sampled gradients. Other methods such as Spatially Varying Covariates models (SVC; Gelfand et al., 2003) could be used to better account for habitat structure complexity across spatial gradients (Thorson et al., 2023).

Previous studies have shown that unaccounted variations in species availability, considering a constant detection probability or unmodelled variations, could lead to substantial bias in estimated abundance (Link et al., 2018; Barker et al., 2018; Duarte, Adams & Peterson, 2018). N-mixture biassed estimations can be linked to non-assessment of the sampled area, where a smaller or greater sampled effective area could lead to under- or overestimation (Kéry & Royle, 2015). In our study, as we relied on distance sampling methods, we define an effective sampled area, based upon collected observation distance, but we also assumed that individuals considered exposed to the sampling (*i.e*., ‘statistically’ available for modelling) could still be undetected due to small species home ranges or plot-specific habitat cover (Chandler, Royle & King, 2011; see Table 1 and Fig. 2, for covariates used to model species detectability and Materials S5 for model formulation). Despite such consideration, for the HDS model, we assumed that detected individuals were homogeneously distributed over the sampled area. Violating this assumption could lead to within-sample variation that needs to be accounted for, otherwise leading to biassed estimates (Mizel, Schmidt & Lindberg, 2018).

Another potential drawback relies on the quantity of data collected through this structured CS scheme. Over the same breeding season, the semistructured scheme EPOC without temporal replicates nor fixed location requirements collected three times the amount of complete checklists as the structured EPOC-ODF scheme, highlighting CS trade-off of scheme standardisation upon data collection over spatial and temporal scales (Devictor, Whittaker & Beltrame, 2010). One way to address this trade-off would be to apply data integration methods mobilising multiple data sources to be used for ecological inferences (Zipkin, Inouye & Beissinger, 2019; Zipkin et al., 2021), either by estimating abundance of less recorded species through trait-based associations (Callaghan, Nakagawa & Cornwell, 2021, 2022; Robinson et al., 2022) or by constructing joint likelihood functions (Fithian et al., 2015; Fletcher et al., 2019).

## Conclusions

Our results suggested an overall lower population size estimate of French common breeding birds obtained from the last French Breeding Bird atlas methodology than from the Hierarchical Distance Sampling modelling used in the present work. Using large-scale datasets from citizen science obtained from standard scheme initiatives allowed us to infer the variation in species abundance, while explicitly modelling the detection process separately from the ecological one. Not accounting for the observation process might have resulted in misleading expert-only estimations of population sizes in the previous atlas, at least for some widespread species not benefitting from exhaustive surveys. In conclusion, our results advocate for more reliance on the use of statistical tools accounting for the detection process, such as hierarchical models, which, in association with large-scale citizen science data, could constitute a standard methodology to estimate reliable abundance from breeding bird atlases or biodiversity surveys deployed at national or geographically broader scales

## Supplemental Information

10.7717/peerj.17889/supp-1 Supplemental Information 1

## Additional Information and Declarations

### Data Availability

The following information was supplied regarding data availability: The data, scripts and additional figures and tables are available at Zenodo: Nabias, J., Barbaro, L., Fontaine, B., Dupuy, J., Couzi, L., Vallé, C., & Lorrilliere, R. (2024). Supplementary information for “Reassessment of French breeding bird population sizes using citizen science and accounting for species detectability” [Data set]. Zenodo. https://doi.org/10.5281/zenodo.11402818.
