# Supplementary material for: Reassessment of French breeding bird population sizes using citizen science and accounting for species detectability
Source: PeerJ. 2024 Aug 27;12:e17889. doi: 10.7717/peerj.17889 (PMC11363910; doi:10.7717/peerj.17889)
Supplement: Supplemental Information 1 [file peerj-12-17889-s001.docx]

**Supplementary information for :**

**“Reassessment of French breeding bird population sizes using citizen science and accounting for species detectability”**

**Jean Nabias^1,2,*^, Luc Barbaro^2,3^, Benoit Fontaine^2,4^, Jérémy Dupuy^1^, Laurent Couzi^1^, Clément Vallé^2^, Romain Lorrillière^2,5^**

^1 :^ *^LPO-BirdLife France, Fonderies Royales – Rochefort Cedex, France^*

^2 :^ *^CESCO, Muséum National d’Histoire Naturelle, CNRS, Sorbonne-University – Paris, France^*

^3 :^ *^Dynafor, INRA-INPT, University of Toulouse – Auzeville, France^*

^4 :^ *^PATRINAT, OFB, Muséum National d’Histoire Naturelle, CNRS – Paris, France^*

*^5 : Centre de Recherches sur la Biologie des Populations d’Oiseaux (CRBPO), MNHN-CNRS-OFB, Paris, France^*

**Summary**

[S1 : Scheme standardisation 2](#_Toc168054865)

[S2 : Species supplementary tables 3](#_Toc168054866)

[Species metadata 3](#_Toc168054867)

[Robustness analysis 8](#_Toc168054868)

[Estimates stability 13](#_Toc168054869)

[S3 : Environmental data formatting 17](#_Toc168054870)

[Selection of reduced PCA axes for habitat cover 17](#_Toc168054871)

[S4 : Distance of observation : barycenter proxy 20](#_Toc168054872)

[S5 : Global overview of HDS framework 21](#_Toc168054873)

[S6 : Species population sizes comparison table 23](#_Toc168054874)

[Comparison ArGeom and HDS 23](#_Toc168054875)

[Estimation over metropolitan France and atlas grid filtering 30](#_Toc168054876)

[HDS species parameter 35](#_Toc168054877)

[Comparison ArGeom and HDS estimates with German population size 39](#_Toc168054878)

[**S7 : Complementary analysis of the comparison** 39](#_Toc168054879)

[References 41](#_Toc168054880)

## S1 : Scheme standardisation

EPOC-ODF sites are selected from a two-stage random sampling. In order to standardise survey effort over France and help local coordinators site assignment, ten 10x10km atlas grids are randomly selected by districts. Each selected grid is subsequently divided as twenty-five 2x2km grids. Ten out of twenty-five 2x2km grids are selected and categorised as "primary" and "secondary" sites (Fig S1.1). Participant birders are tasked to survey over the centroids of five of these selected sites.

Participants are asked to survey designated “primary” sites, but in regards to terrain specificities, inaccessibility or private land, they can survey one of the “secondary” selected sites. Each completed 10x10km grid cell is removed from the sampling pool for future years of the atlas survey.

Observers are asked to survey the selected sites three times during the breeding season, from march to june, with at least 30 days between each visit. Time periods for the first visits - 1st to 31th of march ; second visits - 1st of april to 8th of may and third visit - 9th of march to 15th of june (https://oiseauxdefrance.org/get-involved/epoc-odf).

In high altitude, the first visit, typically in March, isn’t compulsory and the consequent visits are adjustable in regards to snow conditions and site accessibility.


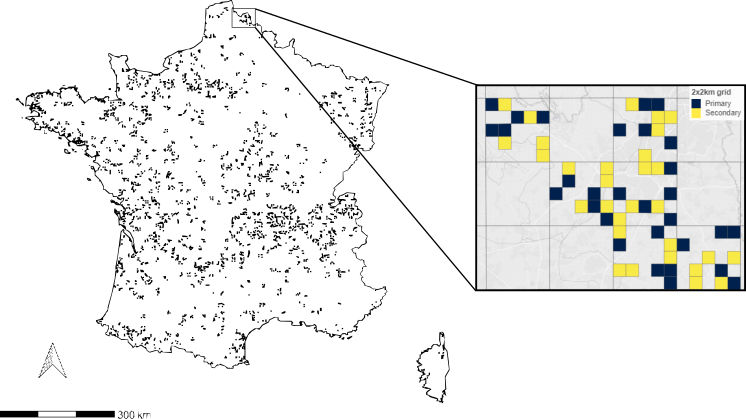


**Figure S1.1** - Schematic of the systematic grid for EPOC-ODF sites.

## S2 : Species supplementary tables

### Species metadata

**Table S2.1** - Species metadata of 63 considered species regrouping FBBS trends between 2012-2023 with their confidence intervals, insignificant trends as well as significant increase and decrease are represented with beige, green and orange colours ; starting and ending phenological periods in julian date ; a priori data filtering based on identifiability of males during survey ad-hoc filters choices; C assessment acceptance.

| **Species** | **FBBS trend (%)**  **2012-2023** | | **Phenological**  **filtering dates** | | ***A priori***  **Male filtering** | **Ĉ** |
| --- | --- | --- | --- | --- | --- | --- |
|  |  |  | **Start** | **End** |  |  |
| Barn Swallow | -13.4 [-20 ; -6.2] |  | 122 | 182 | 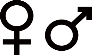 | 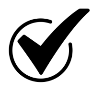 |
| Black Redstart | -10 [-15 ; -4.7] |  | 92 | 152 | 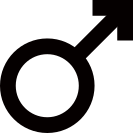 | 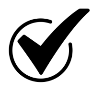 |
| Black Woodpecker | 18.8 [4.8 ; 34.7] |  | 61 | 152 | 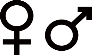 | 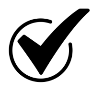 |
| Cetti's Warbler | 132.2 [85.3 ; 190.8] |  | 75 | 152 | 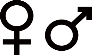 | 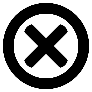 |
| Cirl Bunting | 14.7 [4.2 ; 26.3] |  | 75 | 152 | 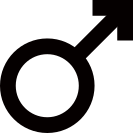 | 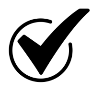 |
| Coal Tit | -48.5 [-55.8 ; -40] |  | 92 | 152 | 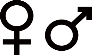 | 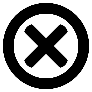 |
| Common Buzzard | -4.8 [-11.1 ; 2] |  | 61 | 152 | 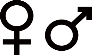 | 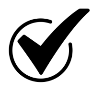 |
| Common Chaffinch | -8.3 [-10.9 ; -5.6] |  | 92 | 152 | 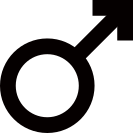 | 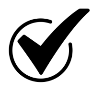 |
| Common Chiffchaff | -1.2 [-4.7 ; 2.3] |  | 92 | 152 | 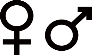 | 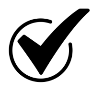 |
| Common Cuckoo | -7.4 [-12.7 ; -1.7] |  | 122 | 182 | 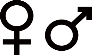 | 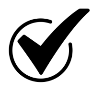 |
| Common Nightingale | -4.7 [-9.8 ; 0.7] |  | 122 | 182 | 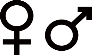 | 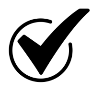 |
| Common Raven | 65.6 [20.7 ; 127.1] |  | 61 | 152 | 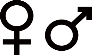 | 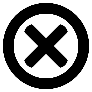 |
| Common Redstart | 14.6 [1.8 ; 29] |  | 122 | 182 | 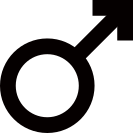 | 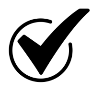 |
| Common Wood-Pigeon | 46.3 [40 ; 52.9] |  | 92 | 152 | 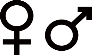 | 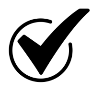 |
| Corn Bunting | 21 [8.2 ; 35.4] |  | 75 | 152 | 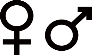 | 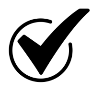 |
| Crested Tit | -28.5 [-38 ; -17.5] |  | 92 | 152 | 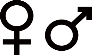 | 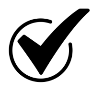 |
| Dunnock | -24.1 [-29.9 ; -17.8] |  | 92 | 152 | 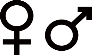 | 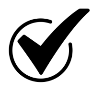 |
| Eurasian Blackbird | -6.8 [-10 ; -3.5] |  | 75 | 152 | 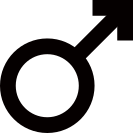 | 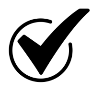 |
| Eurasian Blackcap | 8.8 [5.5 ; 12.1] |  | 106 | 152 | 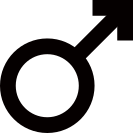 | 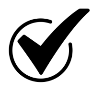 |
| Eurasian Blue Tit | 9.1 [3.4 ; 15] |  | 92 | 152 | 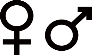 | 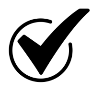 |
| Eurasian Bullfinch | -48 [-56.4 ; -38.1] |  | 75 | 152 | 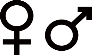 | 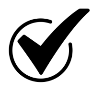 |
| Eurasian Collared-Dove | -4.6 [-9.8 ; 0.8] |  | 75 | 152 | 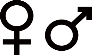 | 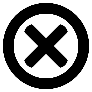 |
| Eurasian Golden Oriole | 15.6 [6.9 ; 25] |  | 122 | 182 | 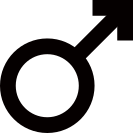 | 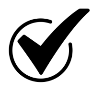 |
| Eurasian Green Woodpecker | -7.8 [-13.3 ; -1.9] |  | 61 | 121 | 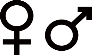 | 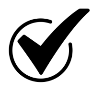 |
| Eurasian Hoopoe | -8.3 [-20.5 ; 5.8] |  | 106 | 167 | 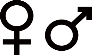 | 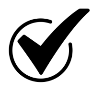 |
| Eurasian Jay | -14.9 [-19.6 ; -9.9] |  | 75 | 167 | 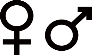 | 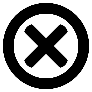 |
| Eurasian Kestrel | 7.3 [-1.8 ; 17.2] |  | 92 | 152 | 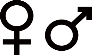 | 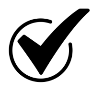 |
| Eurasian Linnet | 34.7 [18.7 ; 52.8] |  | 106 | 167 | 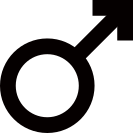 | 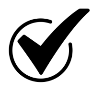 |
| Eurasian Magpie | 11.5 [3.5 ; 20] |  | 61 | 121 | 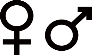 | 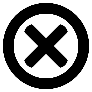 |
| Eurasian Nuthatch | -31.8 [-37.6 ; -25.6] |  | 75 | 152 | 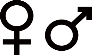 | 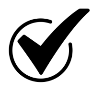 |
| Eurasian Skylark | -9.6 [-14.2 ; -4.7] |  | 92 | 152 | 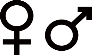 | 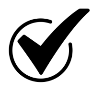 |
| Eurasian Sparrowhawk | -12.6 [-28.5 ; 6.8] |  | 75 | 152 | 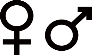 | 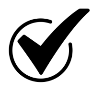 |
| Eurasian Wren | -15 [-18.6 ; -11.1] |  | 92 | 152 | 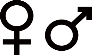 | 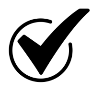 |
| European Goldfinch | 13.7 [4.7 ; 23.6] |  | 106 | 167 | 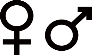 | 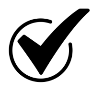 |
| European Greenfinch | -42.5 [-46.8 ; -37.9] |  | 106 | 167 | 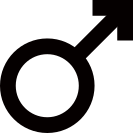 | 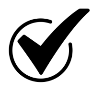 |
| European Robin | 59.8 [52.9 ; 67] |  | 75 | 152 | 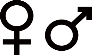 | 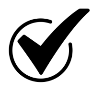 |
| European Serin | -3.7 [-14.1 ; 7.9] |  | 92 | 152 | 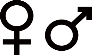 | 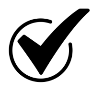 |
| European Stonechat | 23.1 [12.3 ; 34.8] |  | 92 | 152 | 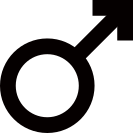 | 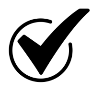 |
| European Turtle-Dove | -25.9 [-32.1 ; -19.1] |  | 136 | 182 | 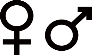 | 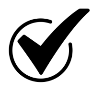 |
| Firecrest | 15.6 [4.6 ; 27.8] |  | 92 | 152 | 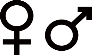 | 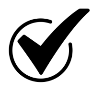 |
| Goldcrest | -46.1 [-53.4 ; -37.7] |  | 92 | 152 | 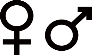 | 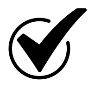 |
| Great Spotted Woodpecker | 15 [8.8 ; 21.4] |  | 61 | 152 | 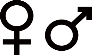 | 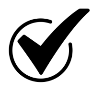 |
| Great Tit | -1.6 [-5.1 ; 2] |  | 92 | 152 | 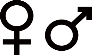 | 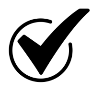 |
| Greater Whitethroat | -5.6 [-11.6 ; 0.9] |  | 122 | 182 | 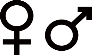 | 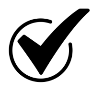 |
| Hawfinch | -17.5 [-33.5 ; 2.4] |  | 75 | 152 | 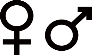 | 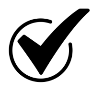 |
| Marsh Tit | -47.7 [-54 ; -40.4] |  | 75 | 152 | 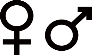 | 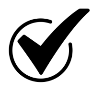 |
| Meadow Pipit | -41.4 [-62.8 ; -7.7] |  | 106 | 167 | 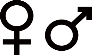 | 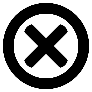 |
| Melodious Warbler | -18 [-24.4 ; -11] |  | 122 | 182 | 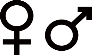 | 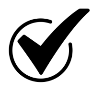 |
| Middle Spotted Woodpecker | 53.1 [21.5 ; 93] |  | 61 | 152 | 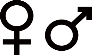 | 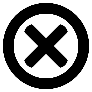 |
| Mistle Thrush | -3.4 [-11.8 ; 5.8] |  | 75 | 152 | 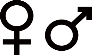 |  |
| Red-backed Shrike | 49.4 [32.3 ; 68.7] |  | 136 | 182 |  |  |
| Ring-necked Pheasant | -1.8 [-12.1 ; 9.7] |  | 61 | 152 |  |  |
| Short-toed Treecreeper | 13.2 [6.3 ; 20.6] |  | 75 | 167 |  |  |
| Song Thrush | 4.7 [-1 ; 10.7] |  | 75 | 152 |  |  |
| Stock Dove | 102.7 [63 ; 152] |  | 75 | 152 |  |  |
| Tree Pipit | -38.9 [-45 ; -32.2] |  | 122 | 182 |  |  |
| Western Bonelli's Warbler | 7.1 [-10.9 ; 28.6] |  | 122 | 182 |  |  |
| Western Yellow Wagtail | -13.7 [-27.5 ; 2.6] |  | 122 | 182 |  |  |
| White Wagtail | -4.9 [-12.9 ; 3.8] |  | 92 | 152 |  |  |
| Willow Warbler | -44.2 [-54.5 ; -31.6] |  | 122 | 182 |  |  |
| Wood Lark | 89.6 [69.4 ; 112.3] |  | 92 | 152 |  |  |
| Yellowhammer | -49.4 [-53.5 ; -44.9] |  | 92 | 152 |  |  |
| Zitting Cisticola | 515 [340.3 ; 758.9] |  | 92 | 167 |  |  |

### Robustness analysis

The robustness analysis consisted of comparing population size estimated using a subset of the calibration dataset. For the period 2021-2022, EPOC-ODF scheme collected 21 662 completed checklists (~79% of total checklists during 2021-2023) over 2874 sites (~74% of total sites surveyed).

Out of the 30 species whose models showed no sign of under/overdispersion (large C), using 2021-2022 EPOC-ODF dataset, nine species had 2021-2023 population size estimates outside of the confidence intervals determined from 2021-2022. 28 species had their confidence intervals shrinked when calibrated from the 2021-2023 dataset, while two species, the Eurasian Linnet (*Carduelis cannabina*) and the Cirl Bunting (*Emberiza cirlus*) had wider confidence intervals using the 2021-2023 dataset. The shrinkage of the confidence interval was estimated by comparing uncertainty ranges (up - low) of the two periods.

**Table S2.2** - Robustness analysis table comparing species population sizes estimated using data collected from 2021-2023 and 2021-2022 EPOC-ODF scheme.

Species which 2021-2023 population size estimated were outside of the confidence interval estimated using 2021-2022 dataset are highlighted in bold. Underlined-italic species names indicate species showing signs of under/overdispersion (Table S2.1). Displayed population sizes correspond to estimations derived from the framework outlined in the present paper after outlier-trimming.

Species without estimation using data collected by the EPOC-ODF scheme 2021-2022 correspond to species detected in less than 150 distinct sites.

| **Species** | **Estimation**  **ODF 2021-2023** | | | **Confidence interval**  **shrinkage** | **Estimation**  **ODF 2021-2022** | | |
| --- | --- | --- | --- | --- | --- | --- | --- |
|  | **low** | **mean** | **up** |  | **low** | **mean** | **up** |
| **Eurasian Blackcap** | 8.6M | 9.5M | 10.5M |  | 12.7M | 14.0M | 15.6M |
| European Robin | 7.7M | 8.5M | 9.4M |  | 7.4M | 8.3M | 9.3M |
| **Common Chaffinch** | 7.2M | 7.9M | 8.8M |  | 9.2M | 10.2M | 11.4M |
| Eurasian Blue Tit | 4.7M | 5.4M | 6.3M |  | 4.5M | 5.3M | 6.3M |
| Great Tit | 4.9M | 5.4M | 6.1M |  | 4.8M | 5.4M | 6.2M |
| **Eurasian Blackbird** | 4.0M | 4.5M | 5.1M |  | 5.2M | 5.8M | 6.5M |
| European Goldfinch | 3.0M | 4.1M | 5.5M | / | / | / | / |
| Common Wood-Pigeon | 3.2M | 3.6M | 3.9M |  | 3.4M | 3.8M | 4.2M |
| Common Chiffchaff | 3.1M | 3.5M | 4.0M |  | 3.2M | 3.7M | 4.3M |
| Eurasian Wren | 3.0M | 3.4M | 3.9M |  | 3.1M | 3.6M | 4.3M |
| Barn Swallow | 1.4M | 2.0M | 2.9M | / | / | / | / |
| Eurasian Linnet | 551.3K | 1.7M | 5.6M |  | 1.7M | 3.2M | 6.0M |
| *Coal Tit* | 3.0M | 1.7M | 5.2M | / | / | / | / |
| Eurasian Skylark | 1.5M | 1.5M | 2.1M | / | / | / | / |
| **Short-toed Treecreeper** | 1.1M | 1.3M | 1.6M |  | 1.6M | 2.0M | 2.5M |
| Firecrest | 962.6K | 1.3M | 1.8M |  | 855.9K | 1.2M | 1.7M |
| *Eurasian Jay* | 1.1M | 1.3M | 1.5M | / | / | / | / |
| *Eurasian Magpie* | 900.9K | 1.2M | 1.8M | / | / | / | / |
| Dunnock | 895.6K | 1.1M | 1.5M |  | 971.1K | 1.3M | 1.8M |
| **Song Thrush** | 966.4K | 1.1M | 1.2M |  | 1.4M | 1.7M | 2.2M |
| White Wagtail | 713.4K | 1.0M | 1.5M |  | 895.6K | 1.6M | 2.8M |
| **Eurasian Nuthatch** | 831.3K | 1.0M | 1.3M |  | 1.3M | 1.9M | 2.6M |
| Goldcrest | 1.3M | 980.6K | 2.7M | / | / | / | / |
| Cirl Bunting | 778.7K | 968.5K | 1.4M |  | 595.3K | 798.0K | 1.1M |
| Black Redstart | 717.1K | 966.4K | 1.3M |  | 834.2K | 1.1M | 1.5M |
| Great Spotted Woodpecker | 752.6K | 930.2K | 1.2M |  | 830.0K | 1.0M | 1.3M |
| *Eurasian Collared-Dove* | 716.6K | 903.1K | 1.2M | / | / | / | / |
| European Greenfinch | 666.0K | 881.2K | 1.2M |  | 604.7K | 823.3K | 1.1M |
| Common Nightingale | 739.8K | 874.9K | 1.0M | / | / | / | / |
| *Meadow Pipit* | 237.2K | 777.3K | 2.9M | / | / | / | / |
| Hawfinch | 422.3K | 747.4K | 1.4M | / | / | / | / |
| European Stonechat | 494.2K | 730.1K | 1.1M |  | 490.3K | 783.9K | 1.3M |
| European Serin | 532.4K | 714.8K | 979.5K | / | / | / | / |
| Marsh Tit | 440.1K | 672.8K | 1.1M | / | / | / | / |
| Greater Whitethroat | 475.9K | 613.5K | 795.1K | / | / | / | / |
| Eurasian Sparrowhawk | 30.2K | 603.5K | 7.3M | / | / | / | / |
| Melodious Warbler | 379.2K | 499.1K | 662.2K | / | / | / | / |
| Crested Tit | 312.2K | 492.5K | 840.5K | / | / | / | / |
| Yellowhammer | 370.4K | 453.6K | 698.9K |  | 414.8K | 518.7K | 815.6K |
| Eurasian Bullfinch | 269.1K | 437.2K | 1.1M | / | / | / | / |
| Red-backed Shrike | 244.1K | 419.9K | 752.0K | / | / | / | / |
| **Common Buzzard** | 270.6K | 367.8K | 502.2K |  | 380.5K | 494.0K | 641.7K |
| European Turtle-Dove | 265.3K | 344.1K | 449.8K | / | / | / | / |
| Mistle Thrush | 296.2K | 341.4K | 417.2K |  | 332.4K | 406.5K | 547.1K |
| Western Bonelli's Warbler | 148.8K | 322.5K | 757.7K | / | / | / | / |
| Eurasian Golden Oriole | 244.9K | 317.7K | 409.3K | / | / | / | / |
| **Eurasian Green Woodpecker** | 263.2K | 316.1K | 380.8K |  | 337.4K | 414.6K | 509.1K |
| Ring-necked Pheasant | 196.1K | 293.4K | 434.3K |  | 176.6K | 274.4K | 420.1K |
| Western Yellow Wagtail | 276.0K | 249.9K | 410.5K | / | / | / | / |
| Tree Pipit | 105.4K | 195.3K | 355.6K | / | / | / | / |
| Corn Bunting | 150.1K | 194.4K | 254.3K | / | / | / | / |
| Wood Lark | 153.1K | 192.9K | 245.2K |  | 135.2K | 201.1K | 305.7K |
| Eurasian Kestrel | 126.1K | 192.5K | 295.9K |  | 148.2K | 233.0K | 368.5K |
| **Common Cuckoo** | 129.8K | 162.3K | 199.9K |  | 770.2K | 1.7M | 3.7M |
| Common Redstart | 39.3K | 129.8K | 283.0K | / | / | / | / |
| Willow Warbler | 34.5K | 113.1K | 341.6K | / | / | / | / |
| *Common Raven* | 26.0K | 104.4K | 340.2K | / | / | / | / |
| *Cetti's Warbler* | 54.3K | 80.6K | 124.2K | / | / | / | / |
| *Zitting Cisticola* | 40.1K | 64.1K | 106.6K | / | / | / | / |
| Black Woodpecker | 26.6K | 44.0K | 71.9K | / | / | / | / |
| Stock Dove | 20.3K | 42.0K | 86.6K | / | / | / | / |
| Eurasian Hoopoe | 25.3K | 41.1K | 64.5K |  | 31.7K | 52.1K | 83.0K |
| *Middle Spotted Woodpecker* | 15.9K | 31.1K | 65.7K | / | / | / | / |

### Estimates stability

We assessed the stability of the estimated population size, over previously known breeding locations [(Issa & Muller, 2015)](https://www.zotero.org/google-docs/?nNJcYg), by measuring the coefficient of variation of the two uncertainty ranges, before and after application of the post-prediction process described in Fig 2.

Out of the 54 species with convenable C values, eight species : Eurasian skylark (Alauda arvensis), Cirl bunting (Emberiza cirlus), Western Yellow wagtail (Motacilla flava), Stock dove (Columba oenas), Goldcrest (Regulus regulus), Crested tit (*Lophophanes cristatus*), Eurasian Bullfinch (*Pyrrhula pyrrhula*) and Yellowhammer (*Emberiza citrinella*) showed large discrepancies (CV > 30%) between the two estimates.

**Table S2.3** - A comparison of the 54 species, with converging HDS model (Table S2.1), population size estimates before and after trimming of extreme predicted abundance values, as shown in Figure 2, and the coefficient of variation (CV) calculated using uncertainty ranges (up - low). The CV was measured using population size estimated over previously known breeding locations.The blue gradient is based on the distribution quantile of the CV values with blue cell shading highlighting species with great discrepancies between the two population sizes estimated.

| **Species** | **Untrimmed** | | | **Outlier-trimmed** | | | **CV**  **(%)** |
| --- | --- | --- | --- | --- | --- | --- | --- |
|  | **low** | **mean** | **up** | **low** | **mean** | **up** |  |
| Eurasian Blackcap | 8.6M | 9.5M | 10.5M | 8.6M | 9.5M | 10.5M | 0.0 |
| European Robin | 7.7M | 8.5M | 9.5M | 7.7M | 8.5M | 9.4M | 2.3 |
| Common Chaffinch | 7.2M | 8.0M | 8.9M | 7.2M | 7.9M | 8.8M | 4.7 |
| Eurasian Blue Tit | 4.7M | 5.4M | 6.4M | 4.7M | 5.4M | 6.3M | 0.8 |
| Great Tit | 4.9M | 5.4M | 6.1M | 4.9M | 5.4M | 6.1M | 0.0 |
| Eurasian Blackbird | 4.0M | 4.7M | 5.6M | 4.0M | 4.5M | 5.1M | 24.1 |
| European Goldfinch | 3.0M | 4.3M | 6.1M | 3.0M | 4.1M | 5.5M | 16.3 |
| Common Wood-Pigeon | 3.2M | 3.6M | 4.0M | 3.2M | 3.6M | 3.9M | 1.5 |
| Common Chiffchaff | 3.1M | 3.5M | 4.0M | 3.1M | 3.5M | 4.0M | 1.0 |
| Eurasian Wren | 3.0M | 3.5M | 4.2M | 3.0M | 3.4M | 3.9M | 16.6 |
| Barn Swallow | 1.4M | 2.1M | 3.2M | 1.4M | 2.0M | 2.9M | 12.7 |
| Eurasian Linnet | 551.3K | 1.7M | 5.7M | 551.3K | 1.7M | 5.6M | 0.6 |
| Eurasian Skylark | 1.5M | 2.3M | 3.7M | 1.5M | 1.5M | 2.1M | 85.0 |
| Short-toed Treecreeper | 1.1M | 1.3M | 1.6M | 1.1M | 1.3M | 1.6M | 0.1 |
| Firecrest | 962.6K | 1.3M | 1.8M | 962.6K | 1.3M | 1.8M | 0.4 |
| Dunnock | 895.6K | 1.2M | 1.8M | 895.6K | 1.1M | 1.5M | 26.5 |
| Song Thrush | 966.4K | 1.1M | 1.3M | 966.4K | 1.1M | 1.2M | 9.7 |
| White Wagtail | 713.4K | 1.0M | 1.5M | 713.4K | 1.0M | 1.5M | 0.0 |
| Eurasian Nuthatch | 831.3K | 1.0M | 1.3M | 831.3K | 1.0M | 1.3M | 0.0 |
| Goldcrest | 1.3M | 12.0M | 197.5M | 1.3M | 980.6K | 2.7M | 139.5 |
| Cirl Bunting | 778.7K | 1.2M | 2.0M | 778.7K | 968.5K | 1.4M | 50.5 |
| Black Redstart | 717.1K | 980.7K | 1.3M | 717.1K | 966.4K | 1.3M | 4.8 |
| Great Spotted Woodpecker | 752.6K | 976.6K | 1.3M | 752.6K | 930.2K | 1.2M | 14.7 |
| European Greenfinch | 666.0K | 886.9K | 1.2M | 666.0K | 881.2K | 1.2M | 1.3 |
| Common Nightingale | 739.8K | 885.5K | 1.1M | 739.8K | 874.9K | 1.0M | 3.5 |
| Hawfinch | 422.3K | 779.2K | 1.6M | 422.3K | 747.4K | 1.4M | 16.7 |
| European Stonechat | 494.2K | 734.9K | 1.1M | 494.2K | 730.1K | 1.1M | 1.9 |
| European Serin | 532.4K | 730.2K | 1.0M | 532.4K | 714.8K | 979.5K | 4.2 |
| Marsh Tit | 440.1K | 728.9K | 1.3M | 440.1K | 672.8K | 1.1M | 21.6 |
| Greater Whitethroat | 475.9K | 613.6K | 795.6K | 475.9K | 613.5K | 795.1K | 0.1 |
| Eurasian Sparrowhawk | 30.2K | 608.0K | 7.6M | 30.2K | 603.5K | 7.3M | 2.9 |
| Melodious Warbler | 379.2K | 500.9K | 665.6K | 379.2K | 499.1K | 662.2K | 0.9 |
| Crested Tit | 312.2K | 549.3K | 1.2M | 312.2K | 492.5K | 840.5K | 33.5 |
| Yellowhammer | 370.4K | 712.6K | 1.8M | 370.4K | 453.6K | 698.9K | 87.3 |
| Eurasian Bullfinch | 269.1K | 1.2M | 12.4M | 269.1K | 437.2K | 1.1M | 123.7 |
| Red-backed Shrike | 244.1K | 434.0K | 818.4K | 244.1K | 419.9K | 752.0K | 8.7 |
| Common Buzzard | 270.6K | 369.6K | 516.2K | 270.6K | 367.8K | 502.2K | 4.1 |
| European Turtle-Dove | 265.3K | 346.5K | 454.3K | 265.3K | 344.1K | 449.8K | 1.7 |
| Mistle Thrush | 296.2K | 373.0K | 474.5K | 296.2K | 341.4K | 417.2K | 27.1 |
| Western Bonelli's Warbler | 148.8K | 334.4K | 852.3K | 148.8K | 322.5K | 757.7K | 10.2 |
| Eurasian Golden Oriole | 244.9K | 318.0K | 410.1K | 244.9K | 317.7K | 409.3K | 0.3 |
| Eurasian Green Woodpecker | 263.2K | 316.2K | 381.2K | 263.2K | 316.1K | 380.8K | 0.2 |
| Ring-necked Pheasant | 196.1K | 294.5K | 437.4K | 196.1K | 293.4K | 434.3K | 0.9 |
| Western Yellow Wagtail | 276.0K | 651.1K | 1.6M | 276.0K | 249.9K | 410.5K | 115.8 |
| Tree Pipit | 105.4K | 197.0K | 365.2K | 105.4K | 195.3K | 355.6K | 2.7 |
| Corn Bunting | 150.1K | 196.2K | 257.8K | 150.1K | 194.4K | 254.3K | 2.3 |
| Wood Lark | 153.1K | 195.6K | 249.9K | 153.1K | 192.9K | 245.2K | 3.5 |
| Eurasian Kestrel | 126.1K | 192.5K | 296.2K | 126.1K | 192.5K | 295.9K | 0.1 |
| Common Cuckoo | 129.8K | 162.3K | 200.0K | 129.8K | 162.3K | 199.9K | 0.1 |
| Common Redstart | 39.3K | 129.8K | 283.0K | 39.3K | 129.8K | 283.0K | 0.0 |
| Willow Warbler | 34.5K | 113.5K | 343.9K | 34.5K | 113.1K | 341.6K | 0.5 |
| Black Woodpecker | 26.6K | 45.7K | 79.5K | 26.6K | 44.0K | 71.9K | 11.0 |
| Stock Dove | 20.3K | 54.9K | 146.5K | 20.3K | 42.0K | 86.6K | 44.0 |
| Eurasian Hoopoe | 25.3K | 41.6K | 65.4K | 25.3K | 41.1K | 64.5K | 1.6 |

## S3 : Environmental data formatting

### Selection of reduced PCA axes for habitat cover

In order to limit the number of variables used to calibrate HDS models and help model convergence (Fig S3.1), we decided to restrain the number of PCA axes for habitat cover variables.

**Figure S3.2** - Variance explained scree plot from 7-class habitat cover PCA.

Explained variance varied from 23.1% to 11.6% on retained six axes (Fig S3.2). After exploration of variable contributions and their associated map projections (Fig S3.3 - S3.4) we decided to use PCA axes 1, 2 and 5, corresponding to 54.71% of explained variance, in order to cover Forets, Urban, Pastures, Annual and Perennial crops gradients. Due to the scheme protocol not targeting wetland birds and habitats, we decided to discard the sixth PCA axis, which showed little to none variation across metropolitan France (Fig S3.4). This specific habitat is monitored through other schemes such as the wetlands network [(Moussy et al., 2022)](https://www.zotero.org/google-docs/?2OzUuU).

**Figure S3.3** - Variable contribution plots for PCA axes. Each plot corresponds to the projection over the PCA axis in order to highlight the most influential variables.

**Figure S3.4** - Projection maps of PCA axes across metropolitan France.

**Table S3.1** - Aggregation table for habitat cover using Theia OSO [(Thierion et al., 2022)](https://www.zotero.org/google-docs/?nDoQtS) at 10m resolution.

| **OSO class** | **Aggregation** | |
| --- | --- | --- |
|  | **Seven-class** | **Three-class** |
| 1 | Urban | Artificial |
| 2 |  |  |
| 3 |  |  |
| 4 |  |  |
| 5 | Annual crops | Open |
| 6 |  |  |
| 7 |  |  |
| 8 |  |  |
| 9 |  |  |
| 10 |  |  |
| 11 |  |  |
| 12 |  |  |
| 14 | Perennial crops |  |
| 15 |  |  |
| 18 | Pastures |  |
| 13 | Grasslands |  |
| 19 |  |  |
| 20 | Minerals / Water bodies |  |
| 21 |  |  |
| 22 |  |  |
| 23 |  |  |
| 16 | Forests | Forests |
| 17 |  |  |

## S4 : Distance of observation : barycenter proxy

For our study, we gathered data using both precise observer locations and approximated observer locations based on the observations barycenter. In the latter case, the observer locations were approximated using observations barycenter. The goal of this appendix is to assess if this approximation could have an effect on a posteriori measured observation distance.

We subsetted our initial dataset using 15 756 lists (i.e. 65% of the global dataset) with registered GPS location of observers. From this subset, we measured the barycenter of observations, in order to measure observation distance given the two types of observer positions, on the same observations. A similar pattern of observation distance distribution emerges between observer positions types (Fig S4.1A). When we estimate differences such as :

$$\Delta=Distances_{GPS}-Distances_{Barycenter}$$

We can see (Fig S4.1B) that the distribution of observation distance differences is slightly offset from 0, depicted as the solid red line. The average observation distance difference is 8.01 m. This suggests that on average observation distances measured using observers GPS locations are greater than those obtained using observation barycenters. This average difference should be taken into account, as the lowest possible resolution during the formation of distance bin classes, in order to limit misclassification of distance class while using these two types of distance data. For our study, the lowest resolution used for distance classes was for the Goldcrest (Regulus regulus) with 18m.

**Figure S4.1** - (A) Density plot of a posteriori measured observation distances given observer position being GPS-tracked or approximated as observations barycenter. (B) Distribution of differences between the two observers positions. Solid red line indicates 0 while dotted blue lines correspond to the 2.5% and 97.5% of the distribution.

## S5 : Global overview of HDS framework

Hierarchical Distance sampling (*HDS*) seeks to model spatial variation of abundance while taking account of the detection process (Kéry & Royle, 2015). As Conventional Distance sampling assumes perfect detection (Buckland et al., 2015), *HDS* relaxes this assumption by assessing the probability that the individual is present and available for detection during survey occasions (Nichols et al., 2009) through multiple visits at the same site.

Considering a population following Poisson distribution with mean , at each site i = 1,2,..,I we have the local population $M_{i}$ :

(i) $M_{i}\sim Poisson(\lambda_{i})$ (1.1)

Given multiple visits j (j = 1,2,..,J), at site i, the number of individuals available for detection $N_{i,j}$ follows binomial outcomes from the local population $M_{i}$ with probability of availability $\varphi_{i,j}$ :

(ii) $N_{i,j}\sim Binomial(M_{i},\varphi_{i,j})$ (1.2)

For each site i and visit j, observers measure the distance of observation between themselves and observed individuals. A vector of cell probabilities $\pi_{i,j}$ derived from a detection function *p* (Buckland et al., 2015) assigns probabilities to distinct distance bins. Observation $y_{i,j}$ can then be described as a multinomial outcome given the number of individuals available for detection and its distance :

(iii) $y_{i,j}\sim Multinomial(N_{i,j}, \pi_{i,j})$ (1.3)

We used the secondary candidate set strategy (Fig S2.1) as described in Morin et al. (2020), where each state of the *HDS* is calibrated from primary candidate sets (Table S5.1) while others are kept constant. We averaged predictions from the final candidate set in order to obtain prediction maps and population size estimates.

**Table S5.1** - Ensemble of sub-models tested in the secondary candidate set approach [(Morin et al., 2020)](https://www.zotero.org/google-docs/?broken=8pSOaY). Count column depicts the number of times each sub-processes was included in the final candidate sets (Figure X2 of the main text), across all studied species.

(*) For the hour effort covariate, we used minutes from sunrise estimated from site longitude, latitude and date of lists completion.

| **States** | **Sub-models** | **Counts** |
| --- | --- | --- |
| Detection | ~ Distance to roads | 7 |
|  | ~ Distance to roads + Proportion of artificial lands (100m) | 0 |
|  | ~ Distance to roads + Proportion of open lands (100m) | 28 |
|  | ~ Distance to roads + Proportion of forests (100m) | 37 |
| Availability | ~ Julian date | 26 |
|  | ~ Julian date + Julian date² | 33 |
|  | ~ Hour (*) | 16 |
|  | ~ Hour + Hour² | 19 |
|  | ~ Julian date + Hour | 26 |
|  | ~ Julian date + Julian date² + Hour | 38 |
|  | ~ Julian date + Hour + Hour² | 33 |
|  | ~ Julian date + Julian date² + Hour + Hour² | 43 |
| Abundance | ~ 3 Bioclimatics PCA axis + 3 Habitat cover PCA axis | 63 |

## S6 : Species population sizes comparison table

### Comparison ArGeom and HDS

**Table S6.1** - Results table of the 54 species with converging HDS model and their associated population sizes, in number of breeding pairs either estimated from the number of singing male encountered or the total number of adults divided by two, see table S2.1 for more details.

Underlined-italic red species names (n = 8) correspond to species not used for the estimates comparison due to important variations between untrimmed and outlier-trimmed predictions (see, Table S2.3) not used for the estimates comparison.

Species habitat specialisation is derived from the FBBS [(Godet et al., 2015)](https://www.zotero.org/google-docs/?c8kIwR). Species conservation status is derived from the IUCN red list for birds in France [(UICN France et al., 2016)](https://www.zotero.org/google-docs/?V6owC9). 2012 ArGeom estimates are retrieved from [Issa and Muller (2015)](https://www.zotero.org/google-docs/?hO99xI) population size estimates, while 2023 estimates correspond to their actualization using FBBS trends (Table S2.1). ArGeom uncertainty corresponds to the decimal logarithm of the difference between maximal and minimal estimated population. Shown HDS estimates correspond to the population size over the known breeding area of the 2012 atlas, see Table S6.2 for population size over the full metropolitan France.

Species mean density and availability estimated through HDS correspond to λ and φ states intercepts in natural scales. Significant intercept ($\alpha$ = 0.05) are represented in bold, near significant intercept ($\alpha$ = 0.1) are followed with an (*). Maximal distance of observation, in meters, represents the further distance bin after right-truncation of 5% of extreme distance values.

Additional informations in regards to HDS calibration are indicated, such as the chosen key function (hn : half-normal ; hr : hazard-rate); chosen distribution (P : Poisson; NB = Negative binomial) used for HDS abundance model, and measure of the overdispersion parameter (C).

The relative difference between HDS and ArGeom estimates ($\delta_{mean}$) is represented over three categories : (i) ArGeom estimates greatly inferior to HDS estimates ($\delta_{mean}$< -0.2) ; (ii) equivalent and (iii) ArGeom estimates greatly superior to HDS estimates ($\delta_{mean}$> 0.2) of ArGeom population size estimations in red,beige and green cell shading respectively. ArGeom midpoints and HDS estimated population size are expressed in pairs.

| **Species** | **Habitat**  **specialization** | **Conservation**  **status** | **ArGeom** | | | **HDS** | | | | | | | | **Ĉ** | **δ_mean_**  **(%)** | |
| --- | --- | --- | --- | --- | --- | --- | --- | --- | --- | --- | --- | --- | --- | --- | --- | --- |
|  |  |  | **Midpoints** | | **Range**  **uncertainty** | **Estimation** | | | **Mean**  **density**  **(ind/km^2^)** | **Mean**  **detection**  **probability** | **Max**  **distance (m)** | **Key**  **function** | **Mixture** |  |  |  |
|  |  |  | **2012** | **2023** |  | **low** | **mean** | **up** |  |  |  |  |  |  |  |  |
| Eurasian Blackcap | Generalist | LC | 6.5M | 7.1M | 6.51 | 8.6M | 9.5M | 10.5M | **16.21** | 1.00 | 210 | hr | P | 1.01 | −0.29 |  |
| European Robin | Forest | LC | 4.5M | 7.2M | 6.68 | 7.7M | 8.5M | 9.4M | **27.51** | **0.76** | 170 | hn | P | 1.01 | −0.17 |  |
| Common Chaffinch | Generalist | LC | 9.0M | 8.3M | 6.56 | 7.2M | 7.9M | 8.8M | **12.99** | 1.00 *^(*)^* | 225 | hr | P | 1.02 | 0.04 |  |
| Eurasian Blue Tit | Generalist | LC | 3.2M | 3.5M | 6.21 | 4.7M | 5.4M | 6.3M | **18.89** | **1.00** | 140 | hn | P | 1.01 | −0.42 |  |
| Great Tit | Generalist | LC | 5.5M | 5.4M | 6.47 | 4.9M | 5.4M | 6.1M | **17.69** | **0.99** | 210 | hn | P | 1.04 | 0.00 |  |
| Eurasian Blackbird | Generalist | LC | 6.5M | 6.1M | 6.45 | 4.0M | 4.5M | 5.1M | **7.61** | **0.98** | 290 | hr | P | 1.06 | 0.29 |  |
| European Goldfinch | Urban | VU | 1.5M | 1.7M | 6.06 | 3.0M | 4.1M | 5.5M | **12.13** | 1.00 | 125 | hr | P | 1.02 | −0.82 |  |
| Common Wood-Pigeon | Generalist | LC | 2.5M | 3.7M | 6.17 | 3.2M | 3.6M | 3.9M | **12.82** | 1.00 | 295 | hn | P | 1.14 | 0.02 |  |
| Common Chiffchaff | Forest | LC | 3.2M | 3.2M | 6.17 | 3.1M | 3.5M | 4.0M | **12.24** | 1.00 *^(*)^* | 205 | hn | P | 1.00 | −0.09 |  |
| Eurasian Wren | Forest | LC | 4.0M | 3.4M | 6.23 | 3.0M | 3.4M | 3.9M | **10.02** | 1.00 | 180 | hn | P | 1.01 | 0.00 |  |
| Barn Swallow | Urban | NT | 1.3M | 1.2M | 5.89 | 1.4M | 2.0M | 2.9M | **5.23** | 1.00 | 175 | hr | P | 1.20 | −0.51 |  |
| Eurasian Linnet | Farmland | VU | 750.0K | 1.0M | 5.83 | 551.3K | 1.7M | 5.6M | 2.58 *^(*)^* | 0.84 | 140 | hn | NB | 1.17 | −0.53 |  |
| *Eurasian Skylark* | Farmland | NT | 1.6M | 1.5M | 5.80 | 1.5M | 1.5M | 2.1M | **1.99** | 1.00 | 275 | hn | NB | 1.25 | −0.03 |  |
| Short-toed Treecreeper | Forest | LC | 1.2M | 1.4M | 5.90 | 1.1M | 1.3M | 1.6M | **4.66** | 1.00 *^(*)^* | 175 | hn | P | 1.04 | 0.05 |  |
| Firecrest | Forest | LC | 700.0K | 809.2K | 5.67 | 962.6K | 1.3M | 1.8M | **3.77** | 1.00 | 115 | hn | P | 1.01 | −0.46 |  |
| Dunnock | Generalist | LC | 1.5M | 1.1M | 5.88 | 895.6K | 1.1M | 1.5M | **3.15** | **1.00** | 170 | hr | P | 0.96 | 0.02 |  |
| Song Thrush | Forest | LC | 1.5M | 1.6M | 6.02 | 966.4K | 1.1M | 1.2M | **3.70** | **0.67** | 330 | hr | P | 1.10 | 0.36 |  |
| White Wagtail | / | LC | 550.0K | 523.0K | 5.46 | 713.4K | 1.0M | 1.5M | **3.93** | 0.77 | 175 | hr | P | 1.10 | −0.66 |  |
| Eurasian Nuthatch | Forest | LC | 1.5M | 1.0M | 5.83 | 831.3K | 1.0M | 1.3M | **3.07** | 0.45 | 240 | hr | P | 1.10 | 0.00 |  |
| *Goldcrest* | Forest | NT | 650.0K | 350.4K | 5.21 | 1.3M | 980.6K | 2.7M | **0.33** | 1.00 | 90 | hn | P | 0.99 | −0.95 |  |
| *Cirl Bunting* | Farmland | LC | 300.0K | 344.1K | 5.36 | 778.7K | 968.5K | 1.4M | **1.57** | **0.69** | 220 | hr | P | 0.95 | −0.95 |  |
| Black Redstart | Urban | LC | 950.0K | 855.0K | 5.80 | 717.1K | 966.4K | 1.3M | **1.30** | 1.00 | 200 | hr | P | 1.06 | −0.12 |  |
| Great Spotted Woodpecker | Forest | LC | 550.0K | 632.5K | 5.54 | 752.6K | 930.2K | 1.2M | **3.04** | **0.59** | 320 | hn | NB | 1.31 | −0.38 |  |
| European Greenfinch | Urban | VU | 1.5M | 862.5K | 5.76 | 666.0K | 881.2K | 1.2M | **1.45** | **0.94** | 215 | hr | P | 1.12 | −0.02 |  |
| Common Nightingale | Generalist | LC | 750.0K | 714.8K | 5.68 | 739.8K | 874.9K | 1.0M | **2.19** | **1.00** | 305 | hn | P | 1.12 | −0.20 |  |
| Hawfinch | Forest | LC | 80.0K | 66.0K | 4.52 | 422.3K | 747.4K | 1.4M | **2.02** | 0.34 *^(*)^* | 130 | hn | P | 1.01 | −1.68 |  |
| European Stonechat | Farmland | NT | 250.0K | 307.8K | 5.09 | 494.2K | 730.1K | 1.1M | 1.23 *^(*)^* | 1.00 | 160 | hn | P | 1.19 | −0.81 |  |
| European Serin | Urban | VU | 375.0K | 361.1K | 5.38 | 532.4K | 714.8K | 979.5K | **1.62** | 1.00 | 155 | hn | P | 1.02 | −0.66 |  |
| Marsh Tit | Forest | LC | 800.0K | 418.4K | 5.32 | 440.1K | 672.8K | 1.1M | **1.49** | 0.87 | 130 | hr | P | 0.99 | −0.47 |  |
| Greater Whitethroat | Farmland | LC | 1.0M | 944.0K | 5.75 | 475.9K | 613.5K | 795.1K | **2.12** | 1.00 | 185 | hr | P | 1.08 | 0.42 |  |
| Eurasian Sparrowhawk | / | LC | 47.3K | 41.3K | 3.87 | 30.2K | 603.5K | 7.3M | 2.07 | **0.05** | 295 | hn | P | 1.13 | −1.74 |  |
| Melodious Warbler | Generalist | LC | 675.0K | 553.5K | 5.57 | 379.2K | 499.1K | 662.2K | **1.73** | 0.25 | 175 | hr | P | 1.08 | 0.10 |  |
| *Crested Tit* | Forest | LC | 450.0K | 321.8K | 5.33 | 312.2K | 492.5K | 840.5K | **0.29** | 1.00 | 125 | hn | P | 0.96 | −0.42 |  |
| *Yellowhammer* | Farmland | VU | 750.0K | 379.5K | 5.40 | 370.4K | 453.6K | 698.9K | **0.55** | 1.00 *^(*)^* | 230 | hr | P | 1.09 | −0.18 |  |
| *Eurasian Bullfinch* | Forest | VU | 150.0K | 78.0K | 4.72 | 269.1K | 437.2K | 1.1M | 0.63 | 0.32 *^(*)^* | 120 | hn | P | 1.05 | −1.39 |  |
| Red-backed Shrike | Farmland | NT | 150.0K | 224.1K | 5.17 | 244.1K | 419.9K | 752.0K | 0.85 | 1.00 | 200 | hn | NB | 1.40 | −0.61 |  |
| Common Buzzard | Farmland | LC | 160.0K | 152.3K | 4.28 | 270.6K | 367.8K | 502.2K | **1.37** | **0.37** | 450 | hr | P | 1.01 | −0.83 |  |
| European Turtle-Dove | / | VU | 400.0K | 296.4K | 5.17 | 265.3K | 344.1K | 449.8K | 1.09 | **1.00** | 285 | hr | P | 1.07 | −0.15 |  |
| Mistle Thrush | Forest | LC | 400.0K | 386.4K | 5.29 | 296.2K | 341.4K | 417.2K | **0.78** | 1.00 | 410 | hn | P | 1.13 | 0.12 |  |
| Western Bonelli's Warbler | Forest | LC | 225.0K | 241.0K | 5.21 | 148.8K | 322.5K | 757.7K | **0.17** | **1.00** | 135 | hn | NB | 1.45 | −0.29 |  |
| Eurasian Golden Oriole | Generalist | LC | 150.0K | 173.4K | 5.06 | 244.9K | 317.7K | 409.3K | **0.58** | 1.00 | 435 | hr | P | 1.05 | −0.59 |  |
| Eurasian Green Woodpecker | Generalist | LC | 225.0K | 207.5K | 5.14 | 263.2K | 316.1K | 380.8K | **1.17** | **0.69** | 500 | hr | P | 1.09 | −0.42 |  |
| Ring-necked Pheasant | / | LC | 205.0K | 201.3K | 4.95 | 196.1K | 293.4K | 434.3K | **0.48** | **0.93** | 380 | hn | NB | 1.18 | −0.37 |  |
| *Western Yellow Wagtail* | Farmland | LC | 135.0K | 116.5K | 4.63 | 276.0K | 249.9K | 410.5K | **0.25** | 1.00 | 190 | hn | NB | 1.25 | −0.73 |  |
| Tree Pipit | / | LC | 375.0K | 229.1K | 5.18 | 105.4K | 195.3K | 355.6K | 0.81 | 1.00 | 255 | hr | NB | 1.09 | 0.16 |  |
| Corn Bunting | Farmland | LC | 300.0K | 363.0K | 5.38 | 150.1K | 194.4K | 254.3K | **0.32** | **1.00** | 270 | hr | P | 1.17 | 0.60 |  |
| Wood Lark | Farmland | LC | 140.0K | 265.4K | 5.06 | 153.1K | 192.9K | 245.2K | **0.82** | 1.00 *^(*)^* | 315 | hn | P | 1.16 | 0.32 |  |
| Eurasian Kestrel | Farmland | NT | 76.0K | 81.5K | 4.23 | 126.1K | 192.5K | 295.9K | **0.62** | 0.94 | 310 | hr | P | 1.05 | −0.81 |  |
| Common Cuckoo | Generalist | LC | 225.0K | 208.3K | 5.14 | 129.8K | 162.3K | 199.9K | **0.59** | **1.00** | 645 | hr | P | 1.18 | 0.25 |  |
| Common Redstart | Urban | LC | 120.0K | 137.5K | 4.84 | 39.3K | 129.8K | 283.0K | **0.33** | 0.00 | 250 | hr | NB | 1.18 | 0.06 |  |
| Willow Warbler | Forest | NT | 150.0K | 83.7K | 4.75 | 34.5K | 113.1K | 341.6K | **0.40** | 0.92 | 130 | hr | NB | 1.36 | −0.30 |  |
| Black Woodpecker | Forest | LC | 32.5K | 38.6K | 4.25 | 26.6K | 44.0K | 71.9K | **0.09** | 0.39 | 555 | hn | P | 1.02 | −0.13 |  |
| *Stock Dove* | / | LC | 45.0K | 91.2K | 4.78 | 20.3K | 42.0K | 86.6K | **0.10** | 0.69 | 380 | hn | P | 0.91 | 0.74 |  |
| Eurasian Hoopoe | Farmland | LC | 85.0K | 77.9K | 4.66 | 25.3K | 41.1K | 64.5K | **0.19** | 1.00 | 455 | hr | P | 1.01 | 0.62 |  |

### Estimation over metropolitan France and atlas grid filtering

**Table S6.2** - Table of the 54 studied species with converging HDS model and their associated population sizes.

For the current study, we restricted the area for model prediction in order to correspond to previously known species’ breeding locations from the last atlas [(Issa & Muller, 2015)](https://www.zotero.org/google-docs/?eFHCxR). These estimates are depicted below the “Atlas grid” spanner.

The HDS model approach used in this paper can estimate population size over geographical areas not collected during the sampling process, while assessing possible issues in regards of extrapolation, see Table S2.3 and Fig 3.), allowing estimation of bird population size over metropolitan France, under the “Metropolitan France” spanner.

Population sizes are represented in the number of breeding pairs, for the HDS approach, this number is either estimated from the number of singing male encountered or the total number of adults divided by two, see table S2.1 for more details. Underlined-italic species names (n = 8) correspond to species not used for the estimates comparison due to important variations between untrimmed and outlier-trimmed population size estimates (see, Table S2.3).

| **Species** | **Atlas grid** | | | | | | | **Metropolitan France**  **HDS (2021-2023)** | | |
| --- | --- | --- | --- | --- | --- | --- | --- | --- | --- | --- |
|  | **ArGeom** | | | | **HDS**  **(2021-2023)** | | |  |  |  |
|  | **2012** | | **2023** | |  |  |  |  |  |  |
|  | **low** | **up** | **low** | **up** | **low** | **mean** | **up** | **low** | **mean** | **up** |
| Eurasian Blackcap | 5.0M | 8.0M | 5.4M | 8.7M | 8.6M | 9.5M | 10.5M | 8.8M | 9.7M | 10.7M |
| European Robin | 3.0M | 6.0M | 4.8M | 9.6M | 7.7M | 8.5M | 9.4M | 8.0M | 8.9M | 9.8M |
| Common Chaffinch | 7.0M | 11.0M | 6.4M | 10.1M | 7.2M | 7.9M | 8.8M | 7.4M | 8.1M | 9.0M |
| Eurasian Blue Tit | 2.5M | 4.0M | 2.7M | 4.4M | 4.7M | 5.4M | 6.3M | 4.9M | 5.7M | 6.6M |
| Great Tit | 4.0M | 7.0M | 3.9M | 6.9M | 4.9M | 5.4M | 6.1M | 5.0M | 5.5M | 6.2M |
| Eurasian Blackbird | 5.0M | 8.0M | 4.7M | 7.5M | 4.0M | 4.5M | 5.1M | 4.1M | 4.6M | 5.3M |
| European Goldfinch | 1.0M | 2.0M | 1.1M | 2.3M | 3.0M | 4.1M | 5.5M | 3.2M | 4.3M | 5.8M |
| Common Wood-Pigeon | 2.0M | 3.0M | 2.9M | 4.4M | 3.2M | 3.6M | 3.9M | 3.4M | 3.7M | 4.1M |
| Common Chiffchaff | 2.5M | 4.0M | 2.5M | 4.0M | 3.1M | 3.5M | 4.0M | 3.3M | 3.7M | 4.2M |
| Eurasian Wren | 3.0M | 5.0M | 2.5M | 4.2M | 3.0M | 3.4M | 3.9M | 3.2M | 3.6M | 4.1M |
| Barn Swallow | 900.0K | 1.8M | 779.4K | 1.6M | 1.4M | 2.0M | 2.9M | 1.5M | 2.1M | 3.1M |
| Eurasian Linnet | 500.0K | 1.0M | 673.5K | 1.3M | 551.3K | 1.7M | 5.6M | 633.7K | 2.0M | 6.5M |
| *Eurasian Skylark* | 1.3M | 2.0M | 1.2M | 1.8M | 1.5M | 1.5M | 2.1M | 1.7M | 1.7M | 2.4M |
| Short-toed Treecreeper | 900.0K | 1.6M | 1.0M | 1.8M | 1.1M | 1.3M | 1.6M | 1.2M | 1.4M | 1.7M |
| Firecrest | 500.0K | 900.0K | 578.0K | 1.0M | 962.6K | 1.3M | 1.8M | 1.2M | 1.6M | 2.2M |
| Dunnock | 1.0M | 2.0M | 759.0K | 1.5M | 895.6K | 1.1M | 1.5M | 989.9K | 1.2M | 1.6M |
| Song Thrush | 1.0M | 2.0M | 1.0M | 2.1M | 966.4K | 1.1M | 1.2M | 1.0M | 1.2M | 1.3M |
| White Wagtail | 400.0K | 700.0K | 380.4K | 665.7K | 713.4K | 1.0M | 1.5M | 748.0K | 1.1M | 1.6M |
| Eurasian Nuthatch | 1.0M | 2.0M | 682.0K | 1.4M | 831.3K | 1.0M | 1.3M | 899.3K | 1.1M | 1.4M |
| *Goldcrest* | 500.0K | 800.0K | 269.5K | 431.2K | 1.3M | 980.6K | 2.7M | 2.1M | 1.2M | 3.4M |
| *Cirl Bunting* | 200.0K | 400.0K | 229.4K | 458.8K | 778.7K | 968.5K | 1.4M | 906.3K | 1.1M | 1.6M |
| Black Redstart | 600.0K | 1.3M | 540.0K | 1.2M | 717.1K | 966.4K | 1.3M | 742.6K | 1.0M | 1.4M |
| Great Spotted Woodpecker | 400.0K | 700.0K | 460.0K | 805.0K | 752.6K | 930.2K | 1.2M | 794.9K | 987.9K | 1.3M |
| European Greenfinch | 1.0M | 2.0M | 575.0K | 1.1M | 666.0K | 881.2K | 1.2M | 693.2K | 919.8K | 1.2M |
| Common Nightingale | 500.0K | 1.0M | 476.5K | 953.0K | 739.8K | 874.9K | 1.0M | 806.9K | 955.9K | 1.1M |
| Hawfinch | 60.0K | 100.0K | 49.5K | 82.5K | 422.3K | 747.4K | 1.4M | 422.3K | 747.4K | 1.4M |
| European Stonechat | 200.0K | 300.0K | 246.2K | 369.3K | 494.2K | 730.1K | 1.1M | 558.2K | 832.9K | 1.2M |
| European Serin | 250.0K | 500.0K | 240.8K | 481.5K | 532.4K | 714.8K | 979.5K | 589.8K | 792.9K | 1.1M |
| Marsh Tit | 600.0K | 1.0M | 313.8K | 523.0K | 440.1K | 672.8K | 1.1M | 553.6K | 847.9K | 1.4M |
| Greater Whitethroat | 700.0K | 1.3M | 660.8K | 1.2M | 475.9K | 613.5K | 795.1K | 547.7K | 714.4K | 938.7K |
| Eurasian Sparrowhawk | 43.1K | 51.5K | 37.7K | 45.0K | 30.2K | 603.5K | 7.3M | 37.0K | 725.2K | 8.8M |
| Melodious Warbler | 450.0K | 900.0K | 369.0K | 738.0K | 379.2K | 499.1K | 662.2K | 425.6K | 564.6K | 755.5K |
| *Crested Tit* | 300.0K | 600.0K | 214.5K | 429.0K | 312.2K | 492.5K | 840.5K | 372.8K | 591.7K | 1.0M |
| *Yellowhammer* | 500.0K | 1.0M | 253.0K | 506.0K | 370.4K | 453.6K | 698.9K | 429.5K | 494.5K | 771.7K |
| *Eurasian Bullfinch* | 100.0K | 200.0K | 52.0K | 104.0K | 269.1K | 437.2K | 1.1M | 355.1K | 550.5K | 1.4M |
| Red-backed Shrike | 100.0K | 200.0K | 149.4K | 298.8K | 244.1K | 419.9K | 752.0K | 244.1K | 419.9K | 752.0K |
| Common Buzzard | 150.0K | 170.0K | 142.8K | 161.8K | 270.6K | 367.8K | 502.2K | 281.0K | 383.2K | 523.7K |
| European Turtle-Dove | 300.0K | 500.0K | 222.3K | 370.5K | 265.3K | 344.1K | 449.8K | 295.2K | 383.6K | 504.4K |
| Mistle Thrush | 300.0K | 500.0K | 289.8K | 483.0K | 296.2K | 341.4K | 417.2K | 322.5K | 369.9K | 453.6K |
| Western Bonelli's Warbler | 150.0K | 300.0K | 160.7K | 321.3K | 148.8K | 322.5K | 757.7K | 210.2K | 464.5K | 1.1M |
| Eurasian Golden Oriole | 100.0K | 200.0K | 115.6K | 231.2K | 244.9K | 317.7K | 409.3K | 286.2K | 377.9K | 495.7K |
| Eurasian Green Woodpecker | 150.0K | 300.0K | 138.3K | 276.6K | 263.2K | 316.1K | 380.8K | 274.5K | 330.3K | 398.7K |
| Ring-necked Pheasant | 160.0K | 250.0K | 157.1K | 245.5K | 196.1K | 293.4K | 434.3K | 252.7K | 384.8K | 579.8K |
| *Western Yellow Wagtail* | 110.0K | 160.0K | 94.9K | 138.1K | 276.0K | 249.9K | 410.5K | 353.3K | 385.5K | 715.0K |
| Tree Pipit | 250.0K | 500.0K | 152.8K | 305.5K | 105.4K | 195.3K | 355.6K | 131.2K | 249.4K | 467.0K |
| Corn Bunting | 200.0K | 400.0K | 242.0K | 484.0K | 150.1K | 194.4K | 254.3K | 184.1K | 240.9K | 318.3K |
| Wood Lark | 110.0K | 170.0K | 208.6K | 322.3K | 153.1K | 192.9K | 245.2K | 218.5K | 281.0K | 363.0K |
| Eurasian Kestrel | 68.0K | 84.0K | 73.0K | 90.1K | 126.1K | 192.5K | 295.9K | 130.7K | 200.0K | 308.5K |
| Common Cuckoo | 150.0K | 300.0K | 138.9K | 277.8K | 129.8K | 162.3K | 199.9K | 138.8K | 174.2K | 215.1K |
| Common Redstart | 90.0K | 150.0K | 103.1K | 171.9K | 39.3K | 129.8K | 283.0K | 47.1K | 171.6K | 405.7K |
| Willow Warbler | 100.0K | 200.0K | 55.8K | 111.6K | 34.5K | 113.1K | 341.6K | 49.4K | 178.1K | 604.0K |
| Black Woodpecker | 25.0K | 40.0K | 29.7K | 47.5K | 26.6K | 44.0K | 71.9K | 29.0K | 49.3K | 82.2K |
| *Stock Dove* | 30.0K | 60.0K | 60.8K | 121.6K | 20.3K | 42.0K | 86.6K | 307.4K | 78.1K | 173.3K |
| Eurasian Hoopoe | 60.0K | 110.0K | 55.0K | 100.9K | 25.3K | 41.1K | 64.5K | 27.4K | 46.6K | 78.2K |

### HDS species parameter

**Table S6.3** - Results table of the 54 species with converging HDS model and their associated population sizes, in number of breeding pairs either estimated from the number of singing male encountered or the total number of adults divided by two, see table S2.1 for more details.

Underlined-italic red species names (n = 8) correspond to species not used for the estimates comparison due to important variations between untrimmed and outlier-trimmed treatment (see, Table S2.3) not used for the estimates comparison.

Species mean density and availability estimated through HDS correspond to λ and φ states intercepts in natural scales. Species detectability () and scale parameters, for species using hazard-rate detection functions are on the log scale, with higher values corresponding to higher probability of detecting individuals at a given distance of observation. Significant estimated parameters ($\alpha$ = 0.05) are represented in bold, near significant intercept ($\alpha$ = 0.1) are followed with an (*).

Additional informations in regards to HDS calibration are indicated, such as the chosen key function (hn : half-normal ; hr : hazard-rate); chosen distribution (P : Poisson; NB = Negative binomial) used for HDS abundance model, and measure of the overdispersion parameter (C).

| **Species** | **Mean**  **density**  **(ind/km^2^)** | **Mean**  **detection**  **probability** | **Key**  **function**  **σ parameter** | **Scale**  **parameter** | **Key**  **function** | **Distribution** | **Ĉ** |
| --- | --- | --- | --- | --- | --- | --- | --- |
| Eurasian Blackcap | **16.21** | 1.00 | **4.60** | **1.57** | hr | P | 1.01 |
| European Robin | **27.51** | **0.76** | **4.03** | / | hn | P | 1.01 |
| Common Chaffinch | **12.99** | 1.00 *^(*)^* | **4.66** | **1.56** | hr | P | 1.02 |
| Eurasian Blue Tit | **18.89** | **1.00** | **3.85** | / | hn | P | 1.01 |
| Great Tit | **17.69** | **0.99** | **4.19** | / | hn | P | 1.04 |
| Eurasian Blackbird | **7.61** | **0.98** | **4.82** | **1.56** | hr | P | 1.06 |
| European Goldfinch | **12.13** | 1.00 | **3.35** | **0.91** | hr | P | 1.02 |
| Common Wood-Pigeon | **12.82** | 1.00 | **4.57** | / | hn | P | 1.14 |
| Common Chiffchaff | **12.24** | 1.00 *^(*)^* | **4.28** | / | hn | P | 1.00 |
| Eurasian Wren | **10.02** | 1.00 | **4.20** | / | hn | P | 1.01 |
| Barn Swallow | **5.23** | 1.00 | **3.75** | **1.00** | hr | P | 1.20 |
| Eurasian Linnet | 2.58 *^(*)^* | 0.84 | **3.78** | / | hn | NB | 1.17 |
| *Eurasian Skylark* | **1.99** | 1.00 | **4.63** | / | hn | NB | 1.25 |
| Short-toed Treecreeper | **4.66** | 1.00 *^(*)^* | **4.05** | / | hn | P | 1.04 |
| Firecrest | **3.77** | 1.00 | **3.73** | / | hn | P | 1.01 |
| Dunnock | **3.15** | **1.00** | **3.98** | **1.17** | hr | P | 0.96 |
| Song Thrush | **3.70** | **0.67** | **4.91** | **1.46** | hr | P | 1.10 |
| White Wagtail | **3.93** | 0.77 | **3.66** | **1.03** | hr | P | 1.10 |
| Eurasian Nuthatch | **3.07** | 0.45 | **4.42** | **1.32** | hr | P | 1.10 |
| *Goldcrest* | **0.33** | 1.00 | **3.55** | / | hn | P | 0.99 |
| *Cirl Bunting* | **1.57** | **0.69** | **4.72** | **1.75** | hr | P | 0.95 |
| Black Redstart | **1.30** | 1.00 | **4.35** | **1.40** | hr | P | 1.06 |
| Great Spotted Woodpecker | **3.04** | **0.59** | **4.54** | / | hn | NB | 1.31 |
| European Greenfinch | **1.45** | **0.94** | **4.43** | **1.47** | hr | P | 1.12 |
| Common Nightingale | **2.19** | **1.00** | **4.62** | / | hn | P | 1.12 |
| Hawfinch | **2.02** | 0.34 *^(*)^* | **3.79** | / | hn | P | 1.01 |
| European Stonechat | 1.23 *^(*)^* | 1.00 | **4.10** | / | hn | P | 1.19 |
| European Serin | **1.62** | 1.00 | **3.97** | / | hn | P | 1.02 |
| Marsh Tit | **1.49** | 0.87 | **3.58** | **1.10** | hr | P | 0.99 |
| Greater Whitethroat | **2.12** | 1.00 | **4.46** | **1.52** | hr | P | 1.08 |
| Eurasian Sparrowhawk | 2.07 | **0.05** | **4.40** | / | hn | P | 1.13 |
| Melodious Warbler | **1.73** | 0.25 | **4.55** | **1.56** | hr | P | 1.08 |
| *Crested Tit* | **0.29** | 1.00 | **3.77** | / | hn | P | 0.96 |
| *Yellowhammer* | **0.55** | 1.00 *^(*)^* | **4.56** | **1.34** | hr | P | 1.09 |
| *Eurasian Bullfinch* | 0.63 | 0.32 *^(*)^* | **3.76** | / | hn | P | 1.05 |
| Red-backed Shrike | 0.85 | 1.00 | **4.11** | / | hn | NB | 1.40 |
| Common Buzzard | **1.37** | **0.37** | **4.96** | **1.43** | hr | P | 1.01 |
| European Turtle-Dove | 1.09 | **1.00** | **4.95** | **1.65** | hr | P | 1.07 |
| Mistle Thrush | **0.78** | 1.00 | **4.91** | / | hn | P | 1.13 |
| Western Bonelli's Warbler | **0.17** | **1.00** | **4.20** | / | hn | NB | 1.45 |
| Eurasian Golden Oriole | **0.58** | 1.00 | **5.14** | **1.55** | hr | P | 1.05 |
| Eurasian Green Woodpecker | **1.17** | **0.69** | **5.22** | **1.49** | hr | P | 1.09 |
| Ring-necked Pheasant | **0.48** | **0.93** | **4.81** | / | hn | NB | 1.18 |
| *Western Yellow Wagtail* | **0.25** | 1.00 | **4.40** | / | hn | NB | 1.25 |
| Tree Pipit | 0.81 | 1.00 | **4.68** | **1.53** | hr | NB | 1.09 |
| Corn Bunting | **0.32** | **1.00** | **4.78** | **1.53** | hr | P | 1.17 |
| Wood Lark | **0.82** | 1.00 *^(*)^* | **4.76** | / | hn | P | 1.16 |
| Eurasian Kestrel | **0.62** | 0.94 | **4.51** | **1.05** | hr | P | 1.05 |
| Common Cuckoo | **0.59** | **1.00** | **5.46** | **1.40** | hr | P | 1.18 |
| Common Redstart | **0.33** | 0.00 | **4.71** | **1.53** | hr | NB | 1.18 |
| Willow Warbler | **0.40** | 0.92 | **4.30** | **1.52** | hr | NB | 1.36 |
| Black Woodpecker | **0.09** | 0.39 | **5.15** | / | hn | P | 1.02 |
| *Stock Dove* | **0.10** | 0.69 | **4.89** | / | hn | P | 0.91 |
| Eurasian Hoopoe | **0.19** | 1.00 | **5.14** | **1.41** | hr | P | 1.01 |

### Comparison ArGeom and HDS estimates with German population size

**Table S6.4** - Comparison of population size estimates from ArGeom and HDS approach, see more detail in table S6.1, with German population size extracted from the European Red List of Birds [(BirdLife International, 2021)](https://www.zotero.org/google-docs/?sWBM0n). Estimated German population size relied on point count and territory mapping methods [(Gedeon et al., 2015)](https://www.zotero.org/google-docs/?ykmYXL). For comparable estimates between the two countries, we applied a coefficient computed on the ratio of countries area (~1.54).

| **Species** | **Scientific names** | **German population sizes** | | **German population sizes (area adjusted)** | | ***ArGeom* Midpoint**  **2023** | **HDS**  **mean** | **mean** |
| --- | --- | --- | --- | --- | --- | --- | --- | --- |
|  |  | **low** | **high** | **low** | **high** |  |  |  |
| Eurasian Blackcap | *Sylvia atricapilla* | 4 650 000 | 6 150 000 | 7 174 047 | 9 488 255 | 7 072 000 | 9 495 742 | -0,29 |
| Firecrest | *Regulus ignicapilla* | 1 250 000 | 1 850 000 | 1 928 507 | 2 854 191 | 809 200 | 1 290 838 | -0,46 |
| Eurasian Blue tit | *Cyanistes caeruleus* | 3 250 000 | 4 800 000 | 5 014 119 | 7 405 468 | 3 545 750 | 5 441 758 | -0,42 |
| Common Cuckoo | *Cuculus canorus* | 38 000 | 62 000 | 58 627 | 95 654 | 208 350 | 162 302 | 0,25 |
| Corn Bunting | *Emberiza calandra* | 16 500 | 29 000 | 25 456 | 44 741 | 363 000 | 194 425 | 0,6 |
| Common Whitethroat | *Curruca communis* | 600 000 | 950 000 | 925 683 | 1 465 665 | 944 000 | 613 495 | 0,42 |

**S7 : Complementary** analysis of the **comparison**

For this complementary analysis, we computed mean using *ArGeom* and HDS upper bounds, instead of the midpoint and mean used in the main text (see fig 5 of the main text). After calibration of the same model described in the “Variation of estimated population sizes between the two approaches” section in the main text, we observe the same response pattern of mean across species detection probabilities and *ArGeom* estimated range uncertainty than those obtained in the main text.

Compare to figure 7, in the main text, despite keeping the significant positive effect of *ArGeom* uncertainty to mean variation, we see that, on average (i.e. in terms of average detection probabilities and *ArGeom* uncertainty from the studied species), there is no significant differences between *ArGeom* and HDS upper bounds.

**Figure S7.1** - Results from the PGLMM. (A) Confidence intervals of the model coefficient, parameters, coefficients significantly different from 0 are represented in blue. Marginal effect plots of population size estimates differences (mean).

mean responses are predicted over gradients of focal terms, either species detection probabilities (B) or ArGeom reported uncertainties (C), while other covariates are held constant at their mean. Species detection probabilities and ArGeom uncertainty are represented on their natural scales, after inverse logit and inverse decimal logarithm transformation, respectively. Dot-dash line corresponds to a mean of 0, signifying estimated population sizes convergence by the two approaches, negative and positive values of mean reflect lower and higher population size estimates of ArGeom relative to those obtained using HDS.

## References

BirdLife International (2021) European Red List of Birds. Office of the European Union.

Buckland ST, Rexstad EA, Marques TA, Oedekoven CS (2015) Distance Sampling: Methods and Applications. Springer International Publishing. https://doi.org/10.1007/978-3-319-19219-2

Gedeon K, Grüneberg C, Mitschke A, Sudfeldt C, Eickhorst W, Fischer S, Flade M, Frick S, Geiersberger I, Koop B, Kramer M, Krüger T, Roth N, Ryslavy T, Stübing S (2015) Atlas Deutscher Brutvogelarten: Atlas of German Breeding Birds. Dachverband Deutscher Avifaunisten, Münster, Westf.

Issa N, Muller Y (2015) Atlas des oiseaux de France métropolitaine: Nidification et présence hivernale. DELACHAUX, Paris.

Kéry M, Royle JA (2015) Applied Hierarchical Modeling in Ecology: Analysis of distribution, abundance and species richness in R and BUGS: Volume 1:Prelude and Static Models. Academic Press, Amsterdam ; Boston.

Morin DJ, Yackulic CB, Diffendorfer JE, Lesmeister DB, Nielsen CK, Reid J, Schauber EM (2020) Is your ad hoc model selection strategy affecting your multimodel inference? Ecosphere, 11, e02997. <https://doi.org/10.1002/ecs2.2997>

Moussy C, Gwenaël Q, Gaudard C (2022) COMPTAGE DES OISEAUX D’EAU À LA MI-JANVIER EN FRANCE : Résultats 2022 du comptage Wetlands International. WETLANDS INTERNATIONAL.

Nichols JD, Thomas L, Conn PB (2009) Inferences About Landbird Abundance from Count Data: Recent Advances and Future Directions. In: Modeling Demographic Processes In Marked Populations Environmental and Ecological Statistics. (eds Thomson DL, Cooch EG, Conroy MJ), pp. 201–235. Springer US, Boston, MA. https://doi.org/10.1007/978-0-387-78151-8_9
